# Blended e-learning with handheld ultrasound devices improves practical competence in eFAST: a randomised controlled study

**DOI:** 10.1186/s12909-026-09054-5

**Published:** 2026-03-25

**Authors:** Lukas Alexander Theodor Platte, Thomas Komanek, Carla Davina Grundmann, Franziska Dietrich-Kiep, Jan Wischermann, Ulrich Hermann Frey

**Affiliations:** Department of Anaesthesiology, Intensive Care, Pain and Palliative Medicine, Marien Hospital Herne, Ruhr University Bochum, Hölkeskampring 40, Herne, 44625 Germany

**Keywords:** Blended learning, Point-of-care ultrasound (POCUS), Handheld ultrasound devices, eFAST (extended Focused Assessment with Sonography for Trauma), Undergraduate medical education, Competency-based training, Randomized controlled trial, Objective Structured Assessment of Ultrasound Skills (OSAUS)

## Abstract

**Background:**

Emergency ultrasound, particularly extended Focused Assessment with Sonography in Trauma (eFAST), is increasingly recognised as essential in undergraduate medical education. Although blended learning offers flexibility, its effect on practical skills remains uncertain. This randomised controlled trial examined whether supplementing voluntary handheld ultrasound practice with digital self-study improved eFAST performance compared with digital preparation alone.

**Methods:**

In this randomised controlled trial, 173 medical students were allocated to a control group (digital self-study; *n* = 89) or an intervention group (digital self-study plus voluntary handheld ultrasound practice; *n* = 84). All the students performed a standardised eFAST on a high-fidelity simulator. A blinded rater assessed performance using a modified Objective Structured Assessment of Ultrasound Skills (OSAUS) score (primary outcome). The secondary outcomes were examination duration, complete eFAST achievement, and student feedback. Regression analyses adjusted for potential confounders.

**Results:**

The intervention group achieved a significantly higher modified OSAUS score (median 16.0 vs. 13.0, *p* < 0.001), completed examinations faster (median 247 vs. 370 s, *p* < 0.001), and more frequently performed a complete eFAST (42.9% vs. 23.6%, OR 2.43, 95% CI 1.26–4.67). Adjusted analyses confirmed these associations (OSAUS + 3.29 points; aOR for complete eFAST 2.52; HR for faster completion 4.26; all *p* < 0.01). Student acceptance was high; intervention participants reported longer preparation times (2.0 vs 1.0 h, *p* < 0.001) without additional disadvantages.

**Conclusions:**

Blended e-learning with handheld ultrasound devices significantly improved eFAST training outcomes, including higher performance scores, shorter examination times, and more complete scans. These findings support the integration of handheld ultrasound practices into the undergraduate curriculum.

**Trial registration:**

Not applicable. This study did not involve a health care intervention on patients but focused on educational training in ultrasound techniques for medical students.

**Supplementary Information:**

The online version contains supplementary material available at 10.1186/s12909-026-09054-5.

## Background

Ultrasound is an essential diagnostic tool and is increasingly integrated into undergraduate medical education [1]. Point-of-care ultrasound (POCUS), including the Focused Assessment with Sonography in Trauma (FAST) examination, is particularly valuable because it allows rapid identification of potentially life-threatening conditions at the scene of an accident or in the emergency department [2]. The European Federation of Societies for Ultrasound in Medicine and Biology (EFSUMB) recommends integrating ultrasound systematically into medical curricula, emphasising both theoretical instruction and hands-on training [2]. Early education in FAST/POCUS has been shown to improve skill acquisition and long-term competency among medical students [3, 4], Consequently, national and international curricula, including the German National Competence-Based Learning Objectives for Undergraduate Medical Education (NKLM 2.0), have incorporated ultrasound as a mandatory element [5, 6].

As ultrasound education has expanded, blended learning, which combines face-to-face teaching with e-learning, has become more relevant. It is as effective as traditional teaching and often yields greater knowledge gains [7]. The major strength of this method is the opportunity to use digital self-study which allows flexibility and repetition, while preserving the limited in-person time for interactive, skill-based training [8].

Recent advances in portable ultrasound devices have further expanded teaching possibilities. Handheld “pocket” ultrasound systems are becoming more widely available, cost-effective, and technically reliable [9, 10]. These devices allow students to practice independently and develop psychomotor skills, such as probe handling and hand–eye coordination, before they attend supervised training sessions [10].

Despite these developments, there is still limited and conflicting evidence on the effectiveness of blended learning concepts in ultrasound education. Multiple studies have shown that e-learning modules and simulation can improve knowledge and confidence, but practical examination scores and objective skill assessments are generally similar between blended and traditional methods [11–15]. Against this backdrop, we explicitly isolate the mechanism of structured, self-directed handheld practice in addition to standardised digital preparation to test for gains in competence, efficiency, and scan completeness. Although overall effects on practical performance have been inconsistent, evidence from simulator-based training shows improvements in scanning skills among medical students [16].

Given this mixed evidence, we investigated whether supplementing digital preparation with hands-on self-practice via a handheld ultrasound device improved medical students’ eFAST performance. The primary outcome was the objective performance score, whereas the secondary outcomes included student perceptions and course acceptance.

## Methods

### Study design

We conducted a prospective, parallel group, randomised controlled trial using a superiority framework, comparing two blended learning formats that differed in the preparatory phase: digital self-study alone versus digital self-study supplemented by voluntary handheld ultrasound practice during the anaesthesiology clerkship. Data collection spanned two academic years (March 2022–June 2023) and ended as planned with no changes to the trial protocol, outcomes, or analyses after commencement. Medical students did not participate in the design, conduct, or reporting of the trial. Teaching and assessment were standardised via a structured basic course and a high-fidelity ultrasound simulator.

### Participants and recruitment

**Fig. 1:**
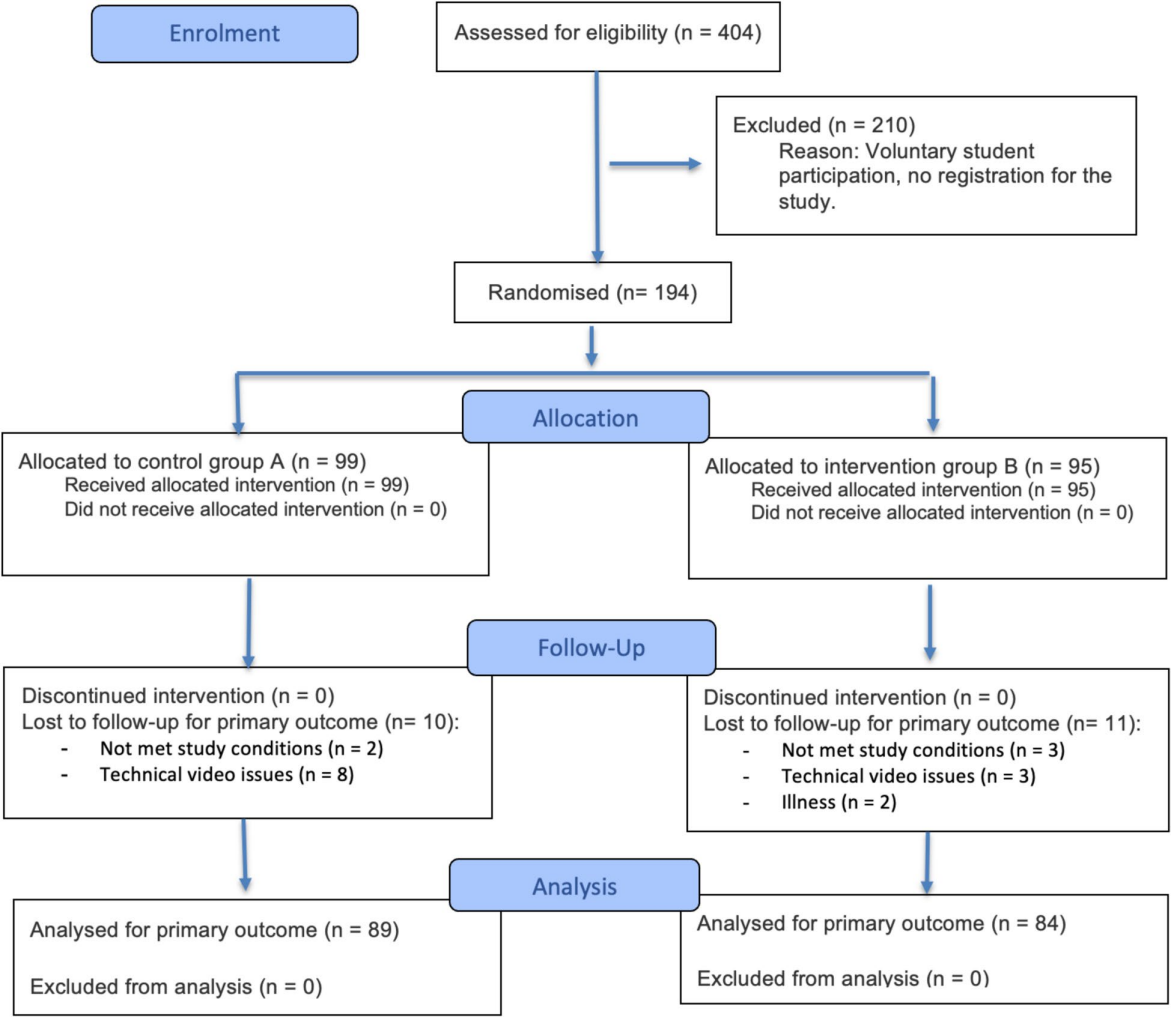
CONSORT 2025 flow diagram of participant progress through the trial (adapted from Hopewell et al.) [17].

Eligible participants were medical students enrolled in the mandatory anaesthesiology clerkships. Recruitment occurred during an information session six weeks before the course. After receiving oral and written study information, the participants provided written informed consent. The inclusion criterion was enrolment in the clerkship and signed informed consent; the exclusion criterion was refusal to participate. Among the 404 students screened, 194 consented; 21 were later excluded for illness or video-recording issues, leaving 173 students for analysis (see Fig. 1, CONSORT flow diagram) [17].

### Randomisation, allocation concealment, and blinding

Randomisation was performed at the clerkship-group level (clusters of 2–3 students predefined by the dean’s office) in a 1:1 ratio to control or intervention. The random sequence was generated via Randomizer.org (see Additional file 3: Tools and Systems) by the study coordination team without stratification or blocking.

Allocation was concealed via central assignment in a password-protected file accessible only to the coordination team. Enrolment was handled by the course administration; assignment was performed exclusively by the coordination team. Course instructors and the outcome assessor were blinded to group allocation but not to the general study aim/hypothesis. The participants and data analysts were not blinded because of the nature of the intervention. Blinding was maintained by separating allocation data from teaching staff and anonymising video recordings for rating.

### Study procedures

**Fig. 2:**
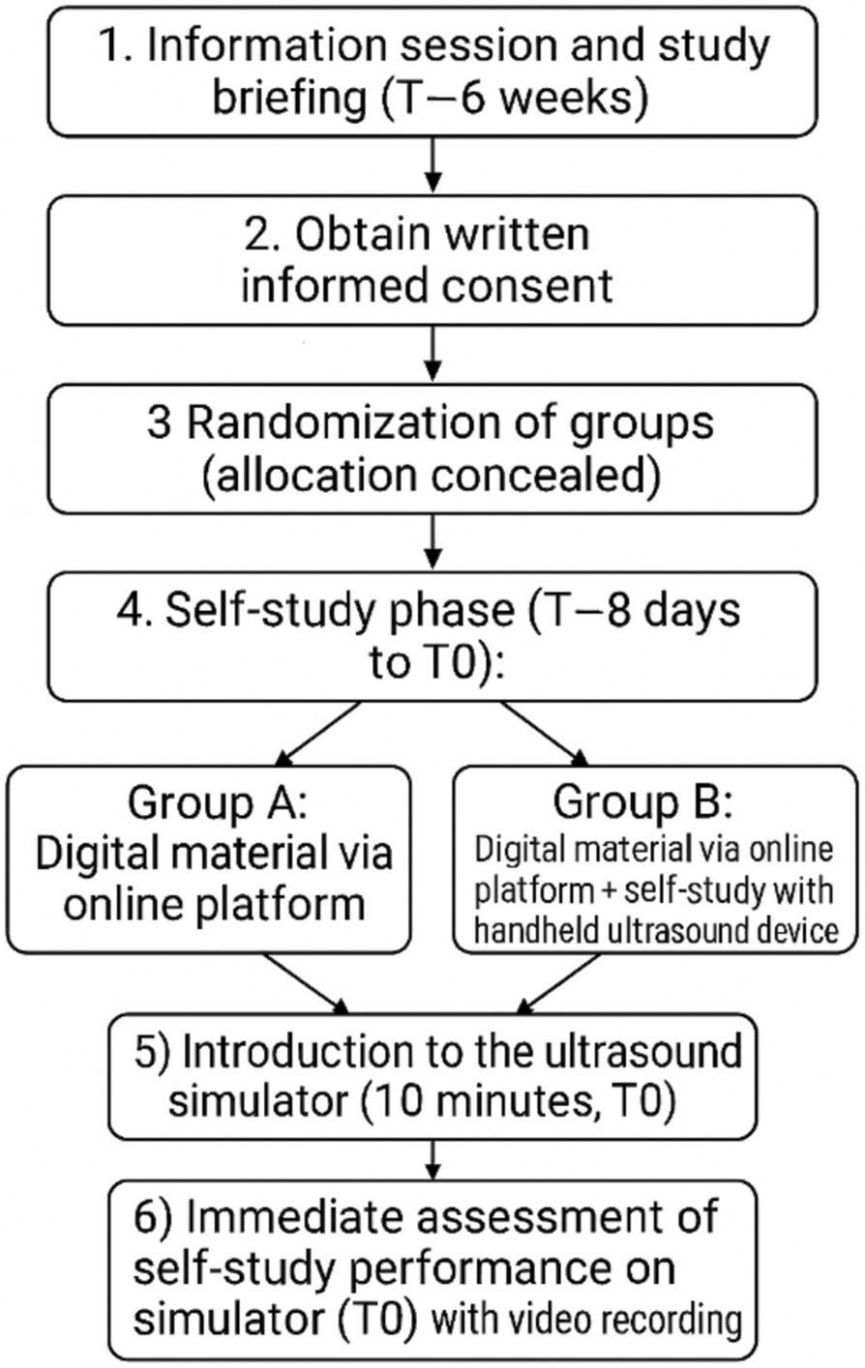
Study flowchart from recruitment to assessment

Six weeks before the course (T–6 weeks), the students attended an information session and provided written informed consent. Clerkship groups were randomised to control or intervention groups with concealed allocation. During the 8-day preparatory phase, all participants accessed standardised digital materials (a presentation and an instructional video) via Moodle (an open-source learning management system at Ruhr University Bochum). The intervention group additionally received a handheld ultrasound device for voluntary self-practice at home. On the day of the examination, all the students received a 10-min standardised introduction to the ultrasound simulator before performing a structured eFAST examination, which was video recorded for blinded evaluation. Named device and platform specifications are provided in Additional file 3: Tools and Systems. The study flowchart is illustrated in Fig. 2.

### Interventions

Both groups received standardised digital preparation; the intervention group additionally had access to a handheld ultrasound device for voluntary practice.

- Digital preparation: Eight days before the course day, all participants were provided with a 31-slide PowerPoint presentation and a 13-min instructional video via the password-protected Moodle platform. The presentation systematically covered the theoretical basics of the eFAST protocol, including scanning points (e.g., pericardium, pleura and abdomen), probe positioning, anatomical landmarks, and image optimisation techniques (e.g., gain adjustment, depth settings). The presentation can be accessed in Additional File 1. The instructional video demonstrated the practical execution of the eFAST examination on a virtual training dummy, synchronised real-time probe positioning with ultrasound images, and addressed common pitfalls and tips for improving image quality. The video is available in Additional File 2.
- Intervention group: In addition to the digital materials, the intervention group provided access to a handheld ultrasound device to enable structured, self-directed practice guided by the standardised digital materials and a brief written device guide. Each randomised clerkship group received one device. A brief device introduction (approximately 5 min) and a written guide were given. Students were encouraged to perform eFAST examinations on themselves or their peers, with a focus on correct probe placement within defined scanning windows. Devices were distributed eight days before the course, and technical support was available via email.
- Common elements: No additional ultrasound teaching occurred outside the study protocol. On the course day, all participants—regardless of group allocation—received a standardised 10-min orientation on the operation of the ultrasound simulator. This briefing focused exclusively on technical aspects (e.g., device settings, interface navigation) and did not include any content-specific guidance. Instructors informally monitored for safety incidents during handheld practice and simulator sessions; no incidents occurred.

### Assessment and examination

The participants were asked to perform a complete eFAST scan on a highly realistic ultrasound simulator and document the findings verbally. The simulator enables realistic representation of abdominal organs and pathological findings (e.g., free fluid, pneumothorax, pericardial tamponade) in various scenarios. Each participant was randomly assigned a simulator case and remained unaware of the underlying pathologies; no clinical vignette was supplied. Ten equally difficult scenarios were used (each with two pathologies; see Table 1). There was no time limit set for the practical examination. The beginning of the time measurement was marked by the first contact of the transducer with the ‘skin’ of the simulator; the timer was stopped when the student declared the examination complete. All classic eFAST examination positions (windows) had to be covered systematically. Examinees were instructed to document all findings verbally (i.e., naming normal and pathological findings).

**Table 1:** Overview of the simulated eFAST scenarios

No	Pathologies in Scenario
1	Pericardial effusion + bilateral pleural effusions
2	Right pleural effusion + free fluid in Morrison’s pouch
3	Pericardial effusion + left pneumothorax
4	Free fluid in Morrison’s pouch + free fluid in Koller’s pouch
5	Right pneumothorax + free fluid in the pelvis
6	Pericardial tamponade + free fluid in Koller’s pouch
7	Right pneumothorax + free fluid in the pelvis
8	Pericardial tamponade + left pleural effusion
9	Left pneumothorax + free fluid in Morrison’s pouch
10	Left pleural effusion + right pneumothorax

Each scenario contained two pathological findings. The scenarios were designed to be of a comparable difficulty level and were randomly assigned to participants during the practical examination

### Outcome measures

The modified OSAUS score (Objective Structured Assessment of Ultrasound Skills) was used for objective performance assessment [18]. The original OSAUS score comprises 17 items in six categories [19, 20]. However, for practicability reasons, the categories ‘Indication’ and ‘Medical decision making’ were omitted in our study. The modified OSAUS score covers five domains: (1) device handling, (2) image optimisation, (3) systematic examination sequence, (4) interpretation of findings, and (5) documentation, each scored from 0 to 5. Predefined deductions were applied for common errors (e.g., incorrect probe orientation or missing one or more eFAST windows). Thus, an error-free examination results in a maximum score of 25 points; the lowest possible score is 0 (see Table 2). The secondary outcomes included examination duration, the completeness of the eFAST scan (i.e. a systematic examination of all probe positions), and the correct recognition of pathologies.

**Table 2:** Modified Objective Structured Assessment of Ultrasound Skills (OSAUS) score, Ruhr University Bochum, 2023–2024

Category	Description	Maximum Points	Typical Deductions
1. Equipment handling	Correct probe selection, proper orientation, correct use of M-mode, safe handling	5	Incorrect orientation (− 1 to − 3), M-mode not activated (− 1), insecure handling (− 1 to − 2)
2. Image optimisation	Proper adjustment of gain and depth (subxiphoid, parasternal, FAST windows)	5	Incorrect gain setting (− 3), depth not adjusted (− 1 to − 3)
3. Systematic approach	All probe positions (subxiphoid, parasternal, FAST I–V) examined in logical sequence	5	Unsystematic examination sequence (− 2), missed position (− 3), multiple positions missed (− 4)
4. Interpretation of findings	All pathologies and normal findings correctly identified	5	Pathology missed (− 2 to − 4), normal finding incorrectly labelled as pathology (− 1 to − 4)
5. Documentation of findings (verbal)	All findings clearly communicated, no laterality errors	5	Pathology not communicated (− 3 to − 4), normal finding not mentioned (− 1), laterality error (− 2 to − 3)

The instrument was adapted from the validated OSAUS framework by omitting two nonpracticable items (“Indication for examination,” “Medical decision making”) and refining the rating guidelines. Each domain was scored from 0 to 5 points, resulting in a maximum total score of 25 points. Typical deductions were predefined to increase transparency and reduce rating variability

### Data collection and rating

**Fig. 3:**
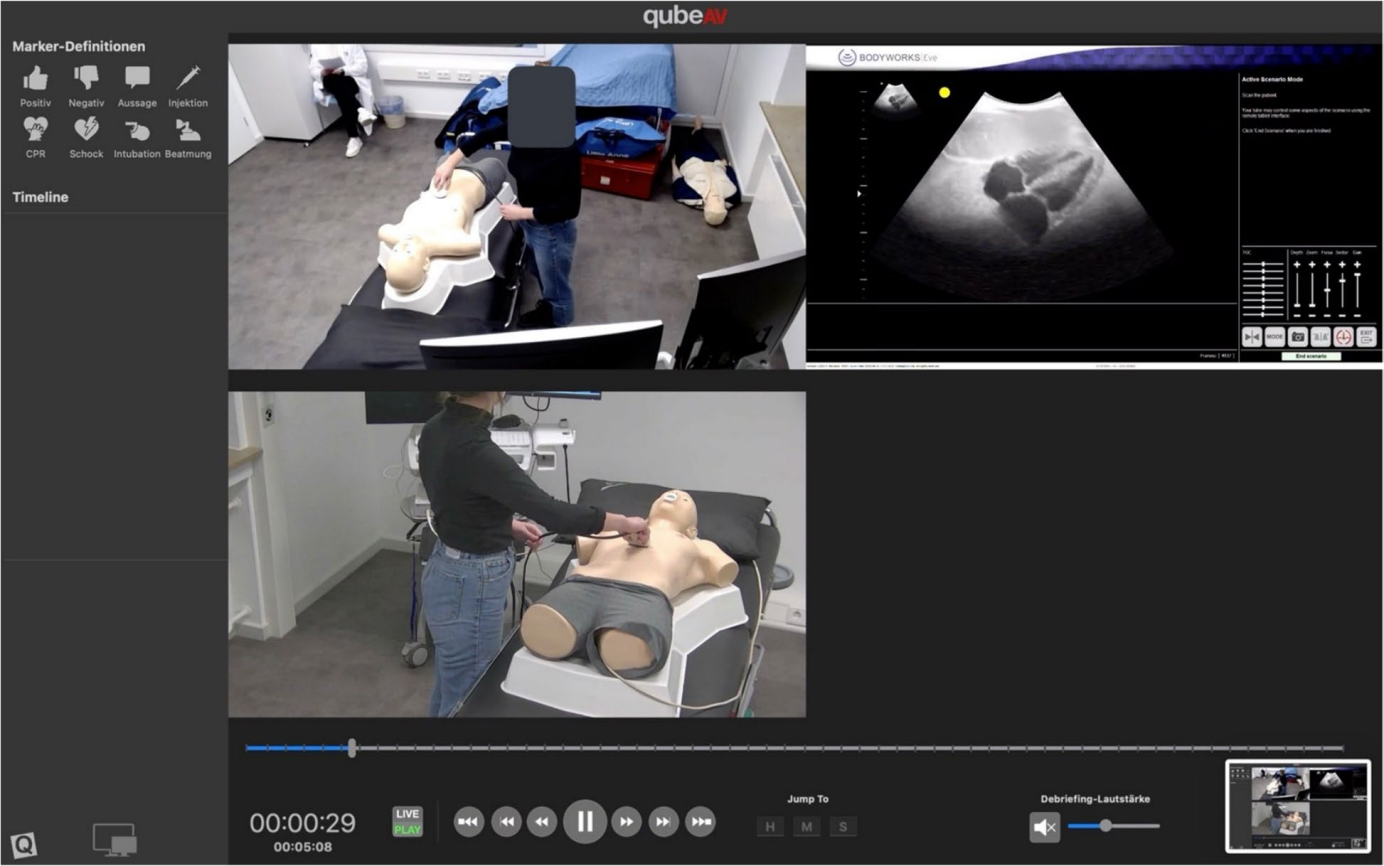
Illustrative screenshot (anonymised) of the video recording during the examination

All examinations were video recorded. An exemplary screenshot is provided in Fig. 3. A qualified rater, an anaesthesiology specialist with board-certified ultrasound training, blinded to group allocation, scored all examinations using the modified OSAUS score. Two trained instructors supervised the assessment sessions to ensure standardisation of the procedure. Scoring followed predefined criteria and typical deductions to increase transparency and reduce variability within the validated OSAUS framework. Baseline data were collected via a standardised questionnaire covering demographics, prior medical training (defined as completion of a healthcare vocational qualification before medical school; nursing/paramedic/other), and prior ultrasound experience (any pre-course exposure: clinical/practicum only or practical exposure plus a voluntary student-tutor fundamentals course, not eFAST), alongside perceived preparedness, satisfaction, self-assessed competence and, for the intervention group, self-reported minutes of handheld practice.

### Statistical analysis

Continuous variables are reported as the means ± SDs or medians [IQRs], depending on normality (Shapiro–Wilk test). t-tests or Mann–Whitney U tests were used for group comparisons, with a rank-biserial effect size (r) and 95% CI. Binary outcomes (complete eFAST) were compared via the absolute risk difference, relative risk, and odds ratio with 95% CI. Holm correction was applied for multiple OSAUS subscales. Exploratory Pearson-Spearman correlations were used to examine the relationships between performance and semester, prior experience, and practice time. Tutor effects were assessed by t-tests. Multivariable regression analysis was conducted with the modified OSAUS total score as the dependent variable and the following independent variables: group allocation (intervention vs. control), age, semester (9 vs. 10), prior ultrasound experience, prior medical training, and tutor assignment. Model assumptions (multicollinearity, normality of residuals, homoscedasticity, and outlier influence) were investigated and met. To further analyse the binary outcome “complete eFAST achieved,” a logistic regression analysis was performed using the same set of covariates. For examination duration, exploratory survival analysis (Cox regression) was considered, with the time to completion as the outcome.

Analyses were conducted with IBM SPSS Statistics 27 (IBM Corp., Armonk, NY, USA) and GraphPad Prism (GraphPad Software LLC, San Diego, CA, USA). The analyses followed a per-protocol approach and included all participants who completed the assessment (*n* = 173).

Missing data were handled by complete-case analysis; there were no missing values for the primary outcome. Primary analyses were prespecified; exploratory correlations and regression models were conducted post hoc.

### Sample size and power

The sample size was determined a priori for a two-arm comparison (control vs. intervention) with a two-sided significance level of α = 0.05 and a power of 80%. On the basis of previous data from undergraduate ultrasound training, we assumed a meaningful performance difference of approximately 2.5 points on the OSAUS scale (range 0–25) with an expected standard deviation of 4.5–5.0 points [21]. This corresponded to a required sample size of approximately 51–63 students per group (total n ≈ 102–126) in an individually randomised design. While randomisation was performed at the clerkship-group level, the calculation was adjusted for cluster effects using a design effect of 1.2–1.3, yielding a target of 122–164 students in total. To account for potential dropouts (10–15% nonattendance or technical data loss), the recruitment goal was set at 180–200 students. Ultimately, 404 students were screened, 194 of whom provided consent and 173 of whom completed the study, thereby exceeding the minimum number required to detect the prespecified differences with adequate power. No interim analyses were planned or conducted, and no stopping guidelines were applied.

## Results

### Baseline characteristics

Among the 194 consenting students, 173 completed the study (*n* = 89 controls, *n* = 84 interventions). Baseline characteristics are presented in Table 3. The mean age was 25.2 ± 3.7 years. Approximately 51% were in the 9th semester, and 49% were in the 10th semester. Tutor assignments were also balanced as well. Overall, 18.5% of the participants had prior medical training, mainly in nursing (56%) and emergency medical services (34%). A total of 69.9% reported prior ultrasound experience (mostly from clinical rotations).

**Table 3:** Baseline characteristics of the study participants (*n* = 173)

Characteristic	Total (*n* = 173)	Control Group A (*n* = 89)	Intervention Group B (*n* = 84)	*p* value
Age, years (Median [IQR])	24 (23–26)	24 (23–25)	24 (23–27)	0.335
Semester				
9th semester, n (%)	89 (51.4%)	44 (49.4%)	45 (53.6%)	
10th semester, n (%)	84 (48.6%)	45 (50.6%)	39 (46.4%)	0.695
Tutor assignment				
Tutor 1, n (%)	87 (50.3%)	45 (50.6%)	42 (50.0%)	
Tutor 2, n (%)	86 (49.7%)	44 (49.4%)	42 (50.0%)	0.944
Prior medical training				
Any prior training, n (%)	32 (18.5%)	12 (13.5%)	20 (23.8%)	0.121
– Paramedic, n (%)	11 (34.4% of trained)	5	6	
– Nursing, n (%)	18 (56.2% of trained)	6	12	
– Other, n (%)	3 (9.4% of trained)	1	2	0.798
Prior ultrasound experience				
Any prior experience, n (%)	121 (69.9%)	59 (66.3%)	62 (73.8%)	0.231
– clinical clerkship, n (%)	73 (60.3% of experienced)	33	40	
– Course + clinical practice, n (%)	48 (39.7% of experienced)	26	22	0.436

Values are presented as medians [IQRs] or numbers (%). *p* values were calculated via χ^2^ test for categorical variables and Mann–Whitney U test for continuous variables

### Primary outcome

**Fig. 4:**
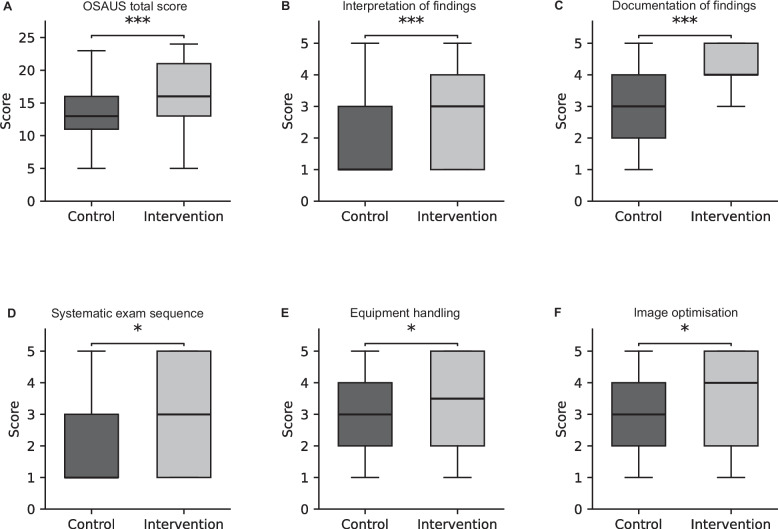
Boxplots showing performance scores across six subscales of the modified OSAUS score for the control and intervention groups. Panels (**A**–**F**) represent: **A** overall modified OSAUS total score, **B** interpretation of findings, **C** documentation of findings, **D** systematic examination sequence, **E** equipment handling, and **F** image optimisation. Boxes indicate the median and interquartile range; whiskers represent the 10th and 90th percentiles. Asterisks denote statistically significant differences between groups (*p* < 0.05, *p < 0.01, **p < 0.001; two-sided Mann–Whitney U test)

The intervention group had a significantly higher median modified OSAUS score than the control group (16.0 [13.0–21.0] vs. 13.0 [11.0–16.0], *p* < 0.001) (Table 4, Fig. 4). This effect was observed across all OSAUS subdomains (interpretation, documentation, handling, optimisation, and sequence; all *p* < 0.05, Holm-adjusted).

**Table 4:** Primary and secondary outcomes of eFAST training

**Variable**		**Control**	**Intervention**	**Effect size r (95% CI)**	***p* value (MWU)**	**Holm-adjusted p**
OSAUS total score		13.0 [11.0–16.0]	16.0 [13.0–21.0]	0.42 (0.29–0.54)	< 0.001	
	Interpretation of findings (Median [IQR])	1.0 [1.0–3.0]	3.0 [1.0–4.0]	0.39 (0.26–0.52)	< 0.001	0.001
	Documentation of findings (Median [IQR])	3.0 [2.0–4.0]	4.0 [4.0–5.0]	0.41 (0.28–0.53)	< 0.001	< 0.001
	Systematic examination sequence (Median [IQR])	1.0 [1.0–3.0]	3.0 [1.0–5.0]	0.21 (0.05–0.36)	0.011	0.032
	Equipment handling (Median [IQR])	3.0 [2.0–4.0]	3.5 [2.0–5.0]	0.18 (0.01–0.35)	0.034	0.038
	Image optimisation (Median [IQR])	3.0 [2.0–4.0]	4.0 [2.0–5.0]	0.20 (0.03–0.36)	0.019	0.038
Examination duration (s)		370.0 [309.0–456.0]	246.5 [186.8–382.8]	− 0.35 (− 0.48 to − 0.21)	< 0.001	
Complete eFAST achieved		23.6%	42.9%	OR = 2.43 (1.26–4.67)	0.007	

Values are presented as medians [IQRs] unless otherwise specified. *p* values were calculated via Mann–Whitney U test; Holm–Bonferroni correction was applied for multiple comparisons; effect sizes are reported as rank-biserial correlations (r)

*OSAUS*  Objective Structured Assessment of Ultrasound Skills, *eFAST*  extended Focused Assessment with Sonography for Trauma, *OR*  odds ratio,  *CI*  confidence interval

### Secondary outcomes

**Fig. 5:**
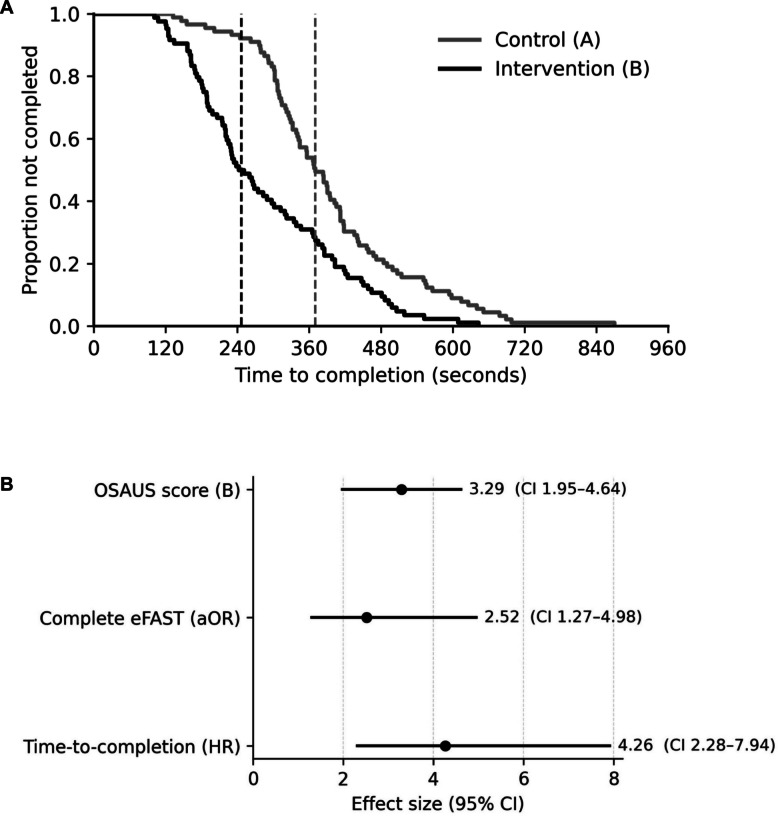
**A** Kaplan–Meier curves for the time-to-completion of the eFAST examination by study group. The intervention group (digital preparation + handheld practice) performed significantly faster than the control group (digital preparation only) (log-rank test, *p* < 0.001). The vertical dashed lines mark median times (Control: 370 s; Intervention: 246.5 s). **B** Forest plot of adjusted effects for the intervention versus the control. The models were adjusted for age, semester, prior ultrasound experience, prior medical training, and tutor assignment. Intervention was associated with higher OSAUS scores (B = 3.29, 95% CI 1.95–4.64), greater odds of complete eFAST (aOR = 2.52, 95% CI 1.27–4.98), and shorter time-to-completion (HR = 4.26, 95% CI 2.28–7.94); OSAUS = Objective Structured Assessment of Ultrasound Skills; aOR = adjusted odds ratio; HR = hazard ratio; CI = confidence interval

The examination duration was significantly shorter in the intervention group than in the control group (median [IQR]: 246 [187–383] vs. 370 [309–456] seconds; Table 4; Fig. 5A). In the intervention group, 42.9% of the students in the intervention group completed a full eFAST (defined as a systematic examination of all relevant probe positions using an ultrasound device), whereas only 23.6% in the control group did (χ^2^ = 7.26, *p* = 0.007). The absolute risk difference was 19.3 percentage points (95% CI 5.5–33.0). The odds ratio for examination completion in the intervention group was 2.43 (95% CI 1.26–4.67; Table 4).

### Multivariable analysis

The adjusted analyses presented in Table 5 confirmed the intervention effect. Linear regression revealed a mean increase of 3.29 OSAUS points for the intervention (95% CI 1.95–4.64, *p* < 0.001). Logistic regression indicated increased odds of completing a full eFAST (aOR 2.52, 95% CI 1.27–4.98, *p* = 0.008). Furthermore, Cox regression demonstrated a significantly shorter time to completion (HR 4.26, 95% CI 2.28–7.94; *p* < 0.001). In the Cox model, only tutor assignment remained a significant predictor (HR 2.03), whereas other covariates were not significant. A forest plot summarising the adjusted effects across models highlights the consistent and pronounced advantage of the intervention group (Fig. 5B).

**Table 5:** Multivariable regression analysis of predictors of eFAST performance

Predictor	OSAUS score (Linear Regression, B [95% CI], p)	Complete eFAST (Logistic Regression, aOR [95% CI], p)	Time-to-completion (Cox Regression, HR [95% CI], p)
Group (Intervention vs. Control)	3.29 [1.95–4.64], < 0.001	2.52 [1.27–4.98], 0.008	4.26 [2.28–7.94], < 0.001
Tutor	1.24 [− 0.14–2.63], 0.079	1.12 [0.58–2.14], 0.742	2.03 [1.13–3.65], 0.018
Age (per year)	0.04 [− 0.23–0.31], 0.760	0.97 [0.88–1.08], 0.629	0.95 [0.85–1.05], 0.269
Semester (10 vs. 9)	0.01 [− 1.34–1.37], 0.985	0.91 [0.48–1.74], 0.776	0.92 [0.69–1.23], 0.572
Prior ultrasound experience (yes vs. no)	1.35 [− 0.13–2.84], 0.074	1.08 [0.58–2.01], 0.809	0.94 [0.68–1.30], 0.716
Prior medical training (yes vs. no)	− 0.24 [− 2.87–2.38], 0.855	0.84 [0.42–1.68], 0.617	0.81 [0.50–1.32], 0.402

Results reported as regression coefficients (B), adjusted odds ratios (aORs), or hazard ratios (HRs) with 95% confidence intervals (CIs) and p values

*OSAUS * Objective Structured Assessment of Ultrasound Skills, *eFAST*  extended Focused Assessment with Sonography for Trauma

### Student feedback

Student feedback showed broad acceptance of the course. Nearly all the participants found the study suitable for exploring approaches to improve teaching quality (A: 98.8%, B: 97.5%, *p* = 0.55). A larger group of control participants felt disadvantaged by the group allocation (27.7% vs. 3.6%, *p* < 0.001). The students in the intervention group reported longer preparation times (median 2.0 vs 1.0 h, *p* < 0.001) and, within that period, self-reported a median of 75 min of handheld practice (IQR 60–120; *n* = 83). The other feedback items did not differ significantly between the groups (see Table 6). No adverse events or harms were observed during the study.

**Table 6:** Group comparisons of feedback items and continuous measures

Item/Variable	A (Yes %/Median [IQR])	B (Yes %/Median [IQR])	n_A	n_B	*p* value	OR (95%‑CI)	RR (95%‑CI)
Prior familiarity with the “blended learning” concept	18/89 (20.2%)	18/84 (21.4%)	89	84	0.8454	0.93 (0.45–1.94)	0.94 (0.53–1.69)
Felt well prepared by the preparatory materials	45/62 (72.6%)	57/65 (87.7%)	62	65	0.0323	0.37 (0.15–0.94)	0.83 (0.69–0.99)
Digital content easy to understand	77/79 (97.5%)	75/78 (96.2%)	79	78	0.6391	1.54 (0.25–9.48)	1.01 (0.96–1.07)
Introductory training in basic technique sufficient	45/66 (68.2%)	56/72 (77.8%)	66	72	0.2037	0.61 (0.29–1.31)	0.88 (0.71–1.08)
Course duration sufficient	49/75 (65.3%)	57/75 (76.0%)	75	75	0.1514	0.60 (0.29–1.21)	0.86 (0.70–1.06)
Post‑course: able to produce good image quality	36/66 (54.5%)	41/62 (66.1%)	66	62	0.1810	0.61 (0.30–1.26)	0.82 (0.62–1.09)
Post‑course: able to recognise pathologies	43/60 (71.7%)	49/63 (77.8%)	60	63	0.4352	0.72 (0.32–1.64)	0.92 (0.75–1.13)
Expectations met	70/78 (89.7%)	69/72 (95.8%)	78	72	0.1529	0.38 (0.10–1.49)	0.94 (0.86–1.02)
Felt disadvantaged by the group allocation	23/83 (27.7%)	3/83 (3.6%)	83	83	< 0.001	10.22 (2.93–35.64)	7.67 (2.39–24.55)
Study suitable to improve teaching quality	80/81 (98.8%)	77/79 (97.5%)	81	79	0.5453	2.08 (0.18–23.39)	1.01 (0.97–1.06)
Ultrasound teaching should be mandatory in the curriculum	88/88 (100%)	84/84 (100%)	88	84	0.9815	1.05 (0.02–53.38)	1.00 (0.98–1.02)
FAST training should be mandatory in the curriculum	87/87 (100%)	84/84 (100%)	87	84	0.9860	1.04 (0.02–52.78)	1.00 (0.98–1.02)
Time spent on preparation (hours, last 8 days)	1.00 [0.50–1.50]	2.00 [1.50–3.00]	89	84	< 0.001	—	—
Relevance of digital preparation (0–100%)	76.00 [63.00–89.00]	71.00 [60.75–88.00]	89	84	0.5159	—	—
Relevance of skills‑based preparation (0–100%)	93.00 [88.00–100.00]	96.00 [91.00–100.00]	89	84	0.1067	—	—
Practice time with mobile devices (minutes, Group B only)	—	75.00 [60.00–120.00]	0	83	—	—	—

Values are presented as numbers and percentages (Yes %) or medians [IQRs]. p values were calculated using χ^2^ test or Fisher’s exact test for categorical variables and Mann–Whitney U test for continuous variables. Effect estimates are shown as odds ratios (ORs) and relative risks (RRs) with 95% confidence intervals (CIs)

## Discussion

In this randomised study, adding handheld ultrasound practice to digital self-study led to better performance than using digital content alone. The intervention group demonstrated higher modified OSAUS scores, shorter examination times, and a higher proportion of complete eFAST scans. These differences had moderate-to-large effect sizes, indicating meaningful educational benefits. Student feedback confirmed high acceptance: nearly all participants found the digital content understandable and the course valuable, whereas the preparation time was significantly longer in the intervention group.

Our findings complement those of prior studies, which reported mixed results for e-learning and blended formats in ultrasound education. Several randomised studies have concluded that e-learning or blended approaches are not only noninferior but also not superior to conventional teaching methods [11–14, 22, 23]. For example, Carstensen et al. reported no difference in musculoskeletal ultrasound skills between e-learning and traditional methods [11]. Similarly, Höhne et al. and Jedwab et al. reported comparable practical outcomes between app-based or online self-training and conventional tuition [12, 13, 22, 23]. In contrast, our results demonstrate clear performance benefits, suggesting that the inclusion of handheld ultrasound practice may provide the missing link that transforms blended learning from equivalence to superiority. Other reports have already hinted at such potential. Jedwab et al. reported that self-directed e-learning for lung POCUS improved test and scanning technique scores compared with bedside teaching, suggesting that digital formats can enhance skills such as image acquisition [13]. Likewise, meta-analyses in health professions also support blended learning for improved knowledge outcomes, although effects on practical skills remain variable [23, 24].

Handheld devices enable independent, repetitive practice that likely strengthens psychomotor and hand–eye coordination. Within a blended model, this plausibly constitutes the active ingredient underlying improved procedural performance [25–29]. Pedagogically, delivering theory online while reserving in-person time for targeted feedback and assessment aligns with contemporary curriculum models [6].

The strengths of this study include its prospective randomised design (minimising bias) [11, 12, 15, 16, 22, 30–32] a clearly defined primary endpoint using the validated OSAUS instrument [11, 15, 20, 22, 30] and standardised simulator environment for all participants, which is consistent with evidence from other domains where simulator-based programs have proven effective [31]. We employed a modified OSAUS score (with predefined criteria) and a blinded rater by using video recordings, enhancing objectivity and reliability.

Limitations include the single-centre design and cluster randomisation at the clerkship-group level (2–3 students), which may limit generalisability to institutions with different organisational and curricular structures and could induce residual intra-cluster correlation. However, teaching and testing were individual and standardised, instructors and the rater were blinded, and our a priori sample size incorporated a design-effect inflation; therefore, any cluster effect was expected to be small. Furthermore, as the cohort comprised late-semester students (9 ^th^−10 ^th^), findings may not extrapolate to earlier learners or to programmes where ultrasound teaching occurs earlier in the curriculum. Additionally, equipment differed between self-practice (handheld) and assessment (simulator); despite essential eFAST functions being available on both, non-identical interfaces and image rendering could have influenced learning transfer and limit generalisability. Furthermore, video-based scoring was performed by a single blinded rater, which should be considered when interpreting the precision of the primary outcome, despite the use of a validated OSAUS rubric with predefined deductions. Although instructors and the outcome assessor were not blinded to the study hypothesis, allocation blinding, anonymised video-based rating, and a predefined OSAUS rubric likely mitigated expectancy effects; nevertheless, residual bias cannot be excluded.

As both participation and handheld practice were voluntary, and practice minutes were captured only by self-report without objective device-level tracking, self-selection bias cannot be excluded (e.g., more motivated or more confident students may have consented and/or practised more), and exposure misclassification is possible. These factors may limit causal attribution between access, practice time, and motivation and temper generalisability to less motivated cohorts. However, randomisation after consent and covariate-adjusted analyses (prior ultrasound experience, prior medical training) help mitigate concerns about internal validity. Furthermore, this single-centre, simulator-based trial did not evaluate long-term skill retention or transfer to patient care; therefore, results should not be extrapolated to clinical performance, and future work will include longitudinal follow-up with bedside assessments. In addition to that, we also did not collect participant gender data, meaning that gender effects could not be analysed.

These limitations highlight avenues for future research: multicentre randomised controlled trials across diverse curricular settings are needed to examine reproducibility and external validity. Stratified randomisation at the individual or small-group level could improve internal validity [6, 22, 33]. Longitudinal studies with follow-up assessments at 6, 12, and 24 months (using frameworks such as OSAUS or I-AIM) should examine skill retention and clinical application [3, 4, 34]. Combining simulation and clinical practice is advisable for retention [3, 4] and cost–benefit analyses are needed to inform large-scale curriculum integration [23, 24, 29]. Finally, studies should also systematically evaluate learner and faculty acceptance, using validated instruments such as the Technology Acceptance Model, to ensure that curricular innovations are sustainable and well received [26, 35, 36].

## Conclusions

Blended e-learning incorporating handheld ultrasound practices significantly improved medical students’ practical competencies in eFAST training. These findings support the integration of handheld ultrasound into undergraduate curricula as part of broader competency-based training in clinical reasoning and procedural skills.

## Supplementary Information

Additional file 1. Presentation of the theoretical basics of the eFAST protocol.

Additional file 2. Instructional video.

Additional file 3. Tools and Systems.

## Data Availability

The data that support the findings of this study, as well as detailed intervention materials (presentation and instructional video), are available in the online Additional file or from the corresponding author upon reasonable request.

## Abbreviations

aOR: Adjusted Odds Ratio
CI: Confidence Interval
CONSORT: Consolidated Standards of Reporting Trials
eFAST: Extended Focused Assessment with Sonography for Trauma
HR: Hazard Ratio
IQR: Interquartile Range
NKLM: Nationaler Kompetenzbasierter Lernzielkatalog Medizin
OSAUS: Objective Structured Assessment of Ultrasound Skills
POCUS: Point-of-care Ultrasound
RR: Relative Risk
SD: Standard Deviation
