# Supplementary material for: Blended e-learning with handheld ultrasound devices improves practical competence in eFAST: a randomised controlled study
Source: BMC Med Educ. 2026 Mar 25;26:580. doi: 10.1186/s12909-026-09054-5 (PMC13063760; doi:10.1186/s12909-026-09054-5)
Supplement: Supplementary file 1 — Additional file 1. Presentation of the theoretical basics of the eFAST protocol. [file 12909_2026_9054_MOESM1_ESM.pdf]

FAST-Sonographie

- FAST-Protokoll: Notfallalgorithmus
- Nachweis/ Ausschluss einer akuten inneren Blutung nach einem Trauma
- Akronym FAST: „Focused Assessment with Sonography for Trauma“
- Sonographische Untersuchungstechniken sind unabdingbar in der Notfallmedizin und bei akutmedizinischen Fragestellungen

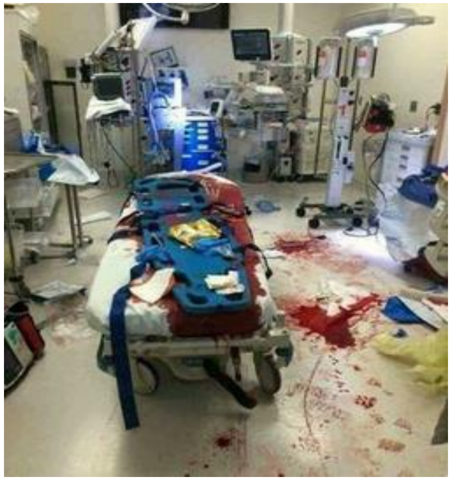

MARIEN HOSPITAL HERNE
MARIEN HOSPITAL HERNE
UK RUB UNIVERSITÄTSKLINIKUM DER RUHR-UNIVERSITÄT BOCHUM
Mitten in der ST. ELISABETH GRUPPE
KATHOLISCHE KLINIKEN RHEIN-RUHR

FAST-Sonographie

- Richtige Schallkopfwahl:
- Der Konvexschallkopf stellt einen Kompromiss dar zwischen ausreichender Schalleindringtiefe, sektorförmiger Erfassung von tiefgelegenen Strukturen und relativ guter Nahauflösung.
- In der fokussierten Abdomensonografie liefert ein 3,5- bis 5-MHz-Konvexschallkopf bei einer Eindringtiefe von ca. 15–20 cm die wichtigsten Informationen.
- Konventionen in der Abdomensonographie:
- Kraniale Strukturen im Sagittalschnitt sowie die rechte Patientenseite im Transversalschnitt sind auf dem Monitor jeweils links randbildend.

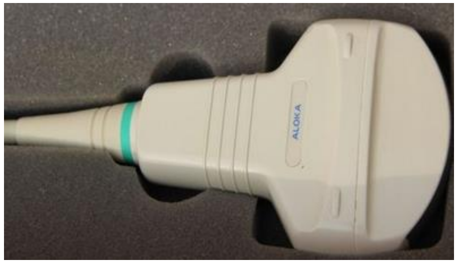

MARIEN HOSPITAL HERNE
MARIEN HOSPITAL HERNE
UK RUB UNIVERSITÄTSKLINIKUM DER RUHR-UNIVERSITÄT BOCHUM
Mitten in der ST. ELISABETH GRUPPE
KATHOLISCHE KLINIKEN RHEIN-RUHR

FAST-Sonographie

- Das FAST-Konzept ist die am weitesten verbreitete sonographische Untersuchungsmethode für fokussierte klinische Fragestellungen.
- Ursprünglich wurde FAST für die thorakoabdominelle Sofortdiagnostik bei polytraumatisierten Patienten entwickelt [5]. Nach heutigem Stand wird FAST in 6 standardisierten Schnitten dazu verwendet, nach freier Flüssigkeit (z.B. bei Blutung) in den Pleuraräumen und tiefen peritonealen Umschlagsfalten zu suchen.
- Da der Patient auf dem Rücken liegt wird das freie Blut, der Schwerkraft folgend, an die tiefsten Punkte fließen

FAST-Sonographie

- Tiefste Punkte (Ansammlung freier Flüssigkeit):
- Pleuraspalt und subphrenisch rechts
- auf der rechten Seite zwischen Leber und Niere (Morison-Pouch)
- Pleuraspalt und subphrenisch links
- auf der linken Seite zwischen Milz und Niere (Koller-Pouch)
- kaudal im Unterbauch (Douglas-Raum: Excavatio rectouterina bei der Frau; Proust-Raum: Excavatio rectovesicalis beim Mann); in der Klinik werden beide Räume vereinfachend oft „Douglas-Raum“ genannt

FAST-Sonographie

- Ergänzung der Untersuchung durch den subxyphoidalen Vierkammer-Blick der transthorakalen Echokardiografie [6]
- Vorteile sind der schnelle Nachweis eines Perikardergusses, die Beurteilung der kardialen Füllung sowie eine orientierende Einschätzung der Pumpfunktion.
- Erweitert werden können diese Schnitte noch (als eFAST (= extended FAST)) durch die Pneumothoraxdiagnostik [7].

FAST-Anlotungspunkte

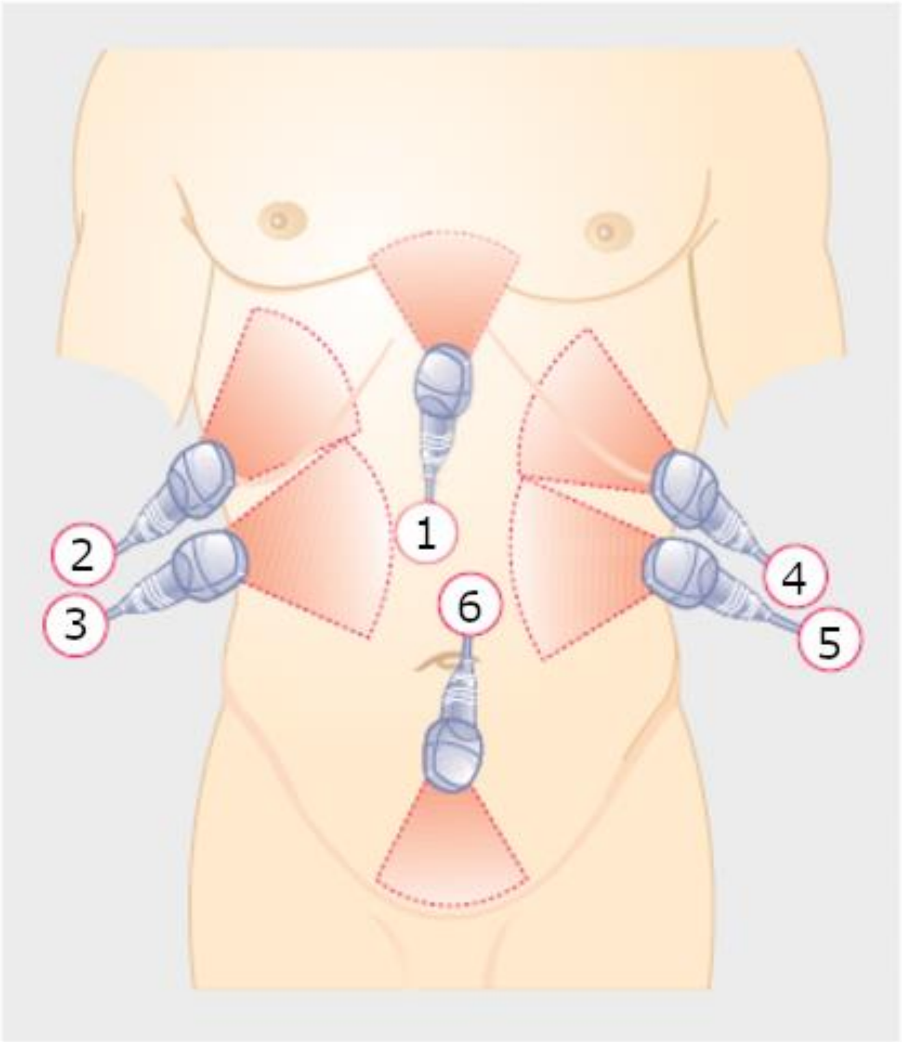

FAST-Anlotungspunkte

- 1: subxyphoidaler Vierkammer-Blick
- 2: lateral-diaphragmaler Längsschnitt rechts: Pleuraraum, subphrenisch;
- 3: lateral-kaudaler Längsschnitt rechts: Morison-Pouch, perihepatisch, Retroperitoneum;
- 4: lateraldiaphragmaler Längsschnitt links: Pleuraraum, subphrenisch, perisplenisch;
- 5: lateral-kaudaler Längsschnitt links: Koller-Pouch, Retroperitoneum;
- 6: medianer Unterbauchschnitt quer/längs: retro- und paravesikal
- Quelle: Helm M, Hauke J, Lampl L. Apparative Diagnostik und Monitoring. In: Scholz J, Sefrin P, Böttiger B et al., Hrsg. Notfallmedizin. 3. Aufl. Stuttgart: Thieme; 2013: 86

MARIEN HOSPITAL HERNE

eFAST – Anlotungspunkte

1. **Subxiphoidaler Vierkammerblick**
2. **Pleura, parasternal rechts**
3. **Pleura, parasternal links**
4. FAST 1: kostodiaphragmaler Winkel re
5. FAST 2: Morison-Pouch
6. FAST 3: kostodiaphragmaler Winkel li
7. FAST 4: Koller-Pouch
8. FAST 5: kleines Becken transv. und sag.

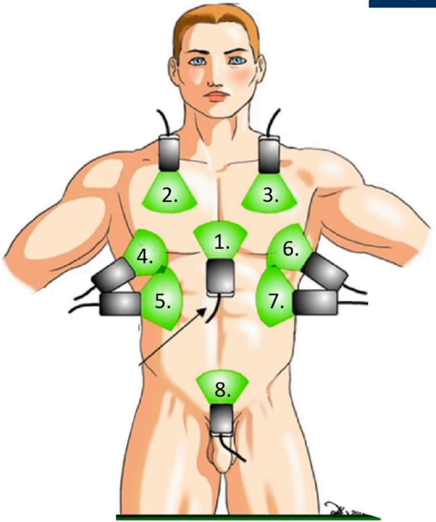

FAST-Fragestellung:

Nr.	Fragestellung	Strukturen	Bezeichnung
1	**Perikarderguss?**<br>**Volumenstatus?**<br>**Pumpfunktion?**	Herz<br>Perikard<br>Leber	Subkostaler Vierkammerblick
2	**Pleuraerguss?**	Leber und Lungenartefakt	Rechter Pleura-Randwinkel
3	**Flüssigkeitssaum zw. Leber und Niere?**	Leber und rechte Niere	Morison-Pouch
4	**Pleuraerguss?**	Milz und Lungenartefakt	Linker Pleura-Randwinkel
5	**Flüssigkeitssaum zw. Milz und Niere?**	Milz und linke Niere	Koller-Pouch
6	**Blasenstatus, Flüssigkeit in Blasenumgebung?**	Harnblase, ggf. Uterus	Douglas-Raum
(7)	**Pneumothorax**	Pneumothorax-Algorithmus	Bat-Sign

MARIEN HOSPITAL HERNE

FAST-Sonographie

- Die FAST-Sonographie gehört sowohl in der Notfallaufnahme, als auch auf der Intensivstation zum festen Standard.
- Sie hilft in zeitkritischen Situationen und bei vitaler Instabilität bei der Detektion freier Flüssigkeit und als Entscheidungshilfe zur frühen Laparotomie [10].
- Der positive FAST-Befund erreicht hierbei eine ähnliche Sensitivität wie die CT-Diagnostik [11].

FAST-Sonographie

- Indikationen für FAST/ eFAST auf der Intensivstation:
  - Verlaufskontrolle nach abdominellem Trauma
  - Verlaufskontrolle nach gefäß- und viszeralchirurgischen Operationen
  - Pleuraerguss- und Aszitesdiagnostik
- Zum schnellen Erlernen und Reproduzieren der FAST-Untersuchung empfiehlt es sich die Reihenfolge einzuhalten. Durch diese Standardisierung können klinisch relevante Befunde innerhalb von wenigen Minuten erhoben werden.
- Klinische Fragestellungen beinhalten die Suche nach Blutungen und die ursächliche Abklärung einer hämodynamischen Instabilität.

FAST-Sonographie

- Bei polytraumatisierten Patienten zählen Verletzungen der Leber, der Milz und der Nieren zu den häufigsten Ursachen für eine intraabdominelle Ansammlung freier Flüssigkeit. Im Thoraxbereich stehen meist Blutgefäßverletzungen und Lungeneinrisse hinter einem Hämatothorax.
- Daneben müssen bei Kreislaufinstabilität eine zusätzlich eine Perikardtamponade und ein Spannungspneumothorax ausgeschlossen werden.
- Die FAST-Positionen 1 und 3 dienen zur Detektion freier Flüssigkeit in den Pleurahöhlen.
- Sonomorphologisch stellt sich Flüssigkeit schwarz (echofrei) dar.

MARIEN HOSPITAL HERNE

FAST-Sonographie

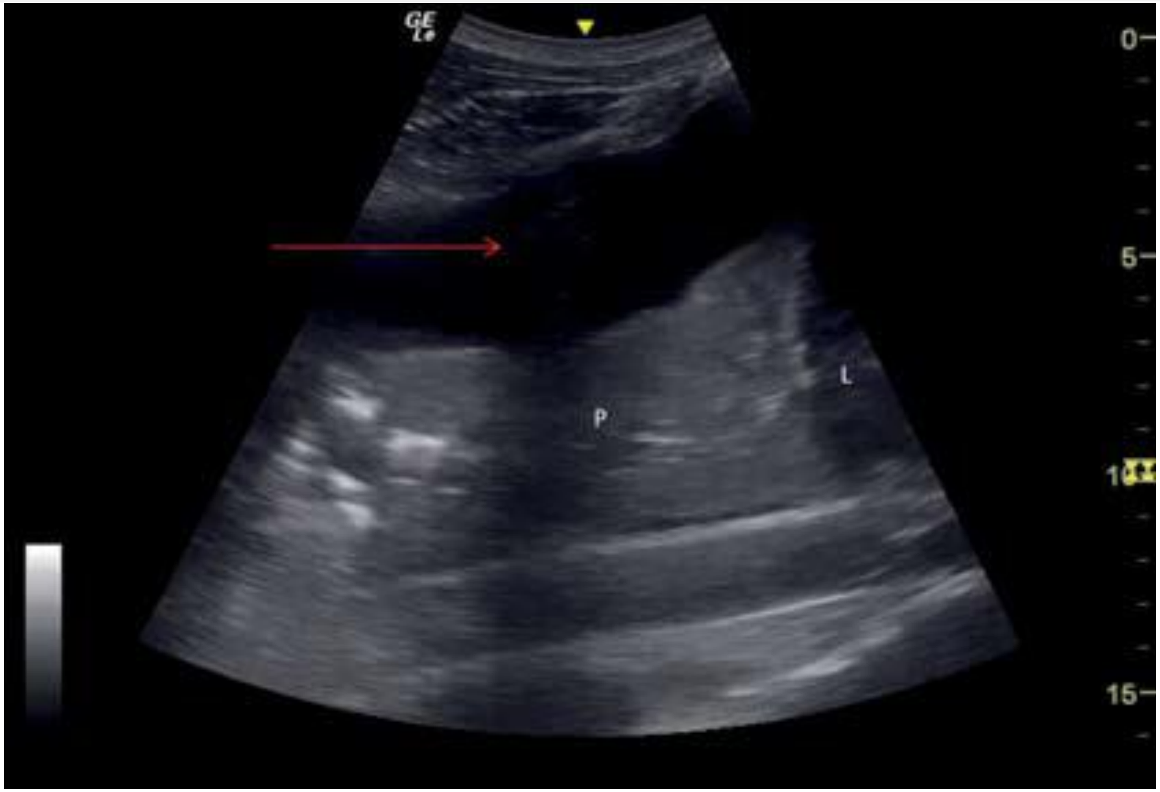

FAST-Position 2 mit positivem Befund. L: Leber, P: Lunge, Pfeil: hypoechogener Erguss; großer Pleuraerguss, der kompressiv auf die Lunge wirkt und zur Atelektasenbildung führt.

Quelle: Schreiber M, Greim CA. Akutsonografie in der Intensivmedizin: Abdomen.
In: Eckart J, Forst H, Briegel J, Hrsg. Intensivmedizin. Kapitel XV – 4.4. Landsberg: ecomed Medizin; 2018: 4.

FAST-Sonographie

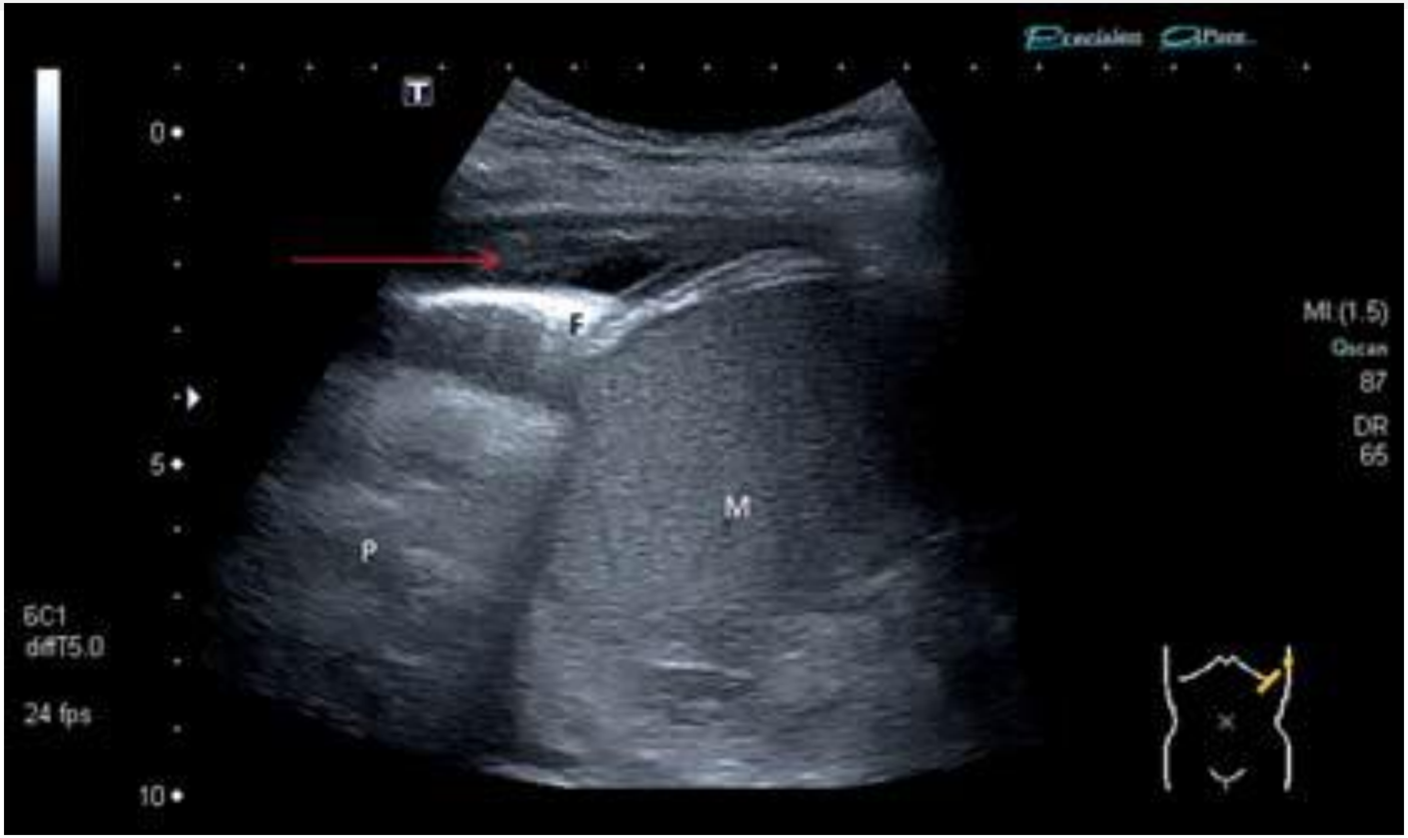

FAST-Position 3 mit positivem Befund. M: Milz, P: Lunge, F: Rippenfraktur, Pfeil: kleiner Erguss.

Quelle: Schreiber M, Greim CA. Akutsonografie in der Intensivmedizin: Abdomen. In: Eckart J, Forst H, Briegel J, Hrsg.Intensivmedizin. Kapitel XV – 4.4. Landsberg: ecomed Medizin; 2018: 4.

FAST-Sonographie: Perikardtamponade

- Unter physiologischen Bedingungen ist die Perikardhöhle nur mit ca. 10–15ml Flüssigkeit gefüllt. Wegen der geringen Dehnbarkeit des Perikards haben größere Flüssigkeitszunahmen in der Perikardhöhle oft massive hämodynamische Auswirkungen.
- Von einer Perikardtamponade spricht man, wenn der Erguss die Füllung des Herzens mit Schockfolge behindert.
- Bei einem hämodynamisch instabilen Patienten ist die Sonographie in der Akutsituation das Verfahren der Wahl, um eine Perikardtamponade auszuschließen.
- Eingestellt wird der subkostale Vierkammerblick, FAST-Position 1

MARIEN HOSPITAL HERNE

FAST-Sonographie: Leberruptur/ Hämatom

MARIEN HOSPITAL HERNE

- Bei abdominellen Traumata, aber auch bei Lebererkrankungen, z.B. HELLP-Syndrom (Haemolysis, elevated Liver Enzymes, low Platelet Count) oder Tumoren, kann es zu Leberrupturen mit Hämatombildung und Kreislaufinstabilität kommen.
- Die FAST-Position 3 eignet sich zur orientierenden Darstellung des rechten Leberlappens und stellt den Raum zwischen kaudaler Leber und dem oberen Nierenpol dar (s. Abb.).
- In diesem sog. Morison-Pouch kann sich nach einem abdominellen Trauma durch die Taschenbildung des Peritoneums freie Flüssigkeit ansammeln. Diese wird in 60–90% der Fälle sonographisch erkannt [14, 15].

MARIEN HOSPITAL HERNE

FAST-Sonographie

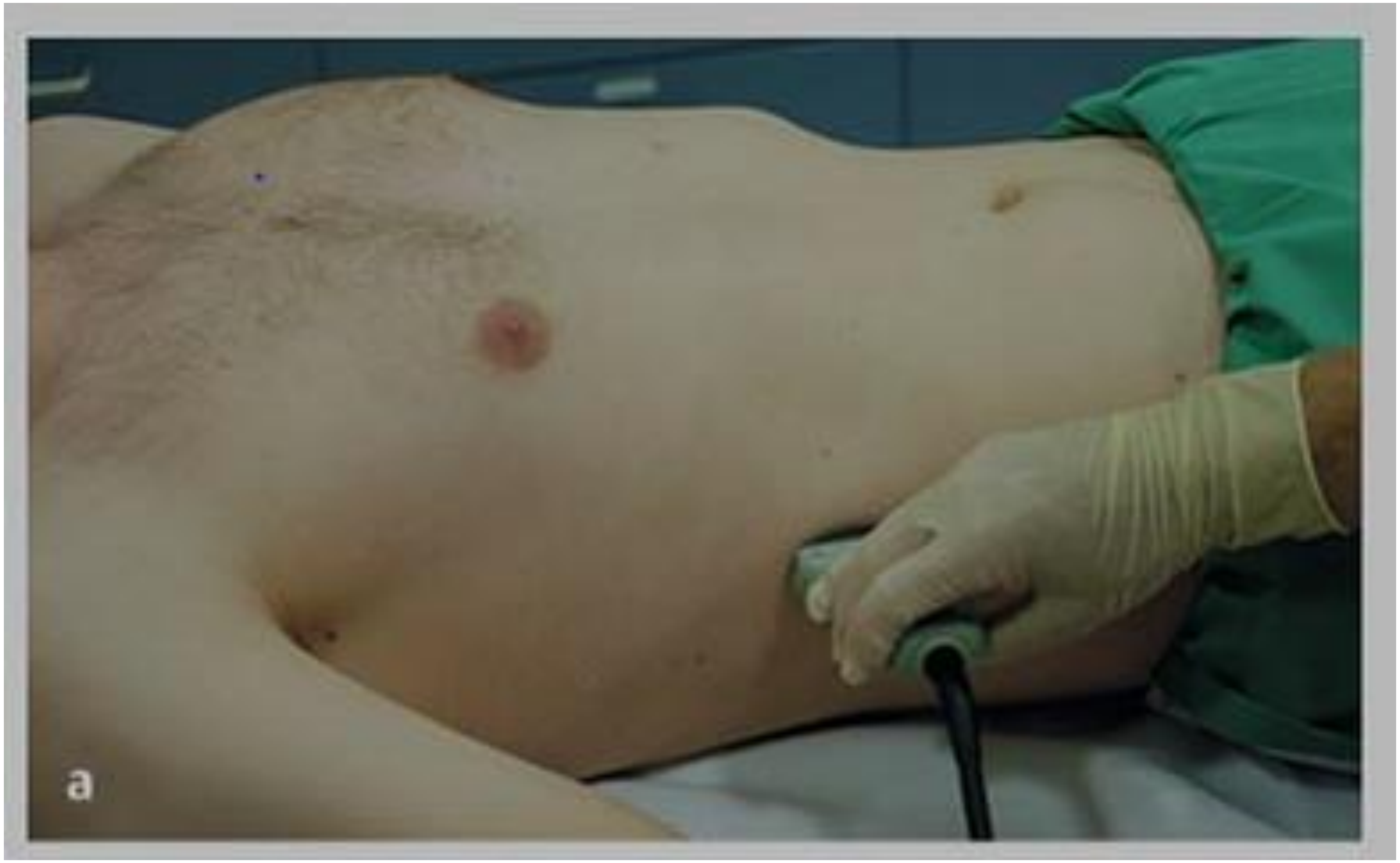

FAST-Position 3: Morison-Pouch. L: Leber, N: Niere. a Darstellung der Schallkopfführung. Quelle: Bord JP et al. Zielorientierte Notfallsonografie bei Traumapatienten (FAST-Protokoll). Dtsch Med Wochenschr 2008; 133: 2646–2648.

FAST-Sonographie

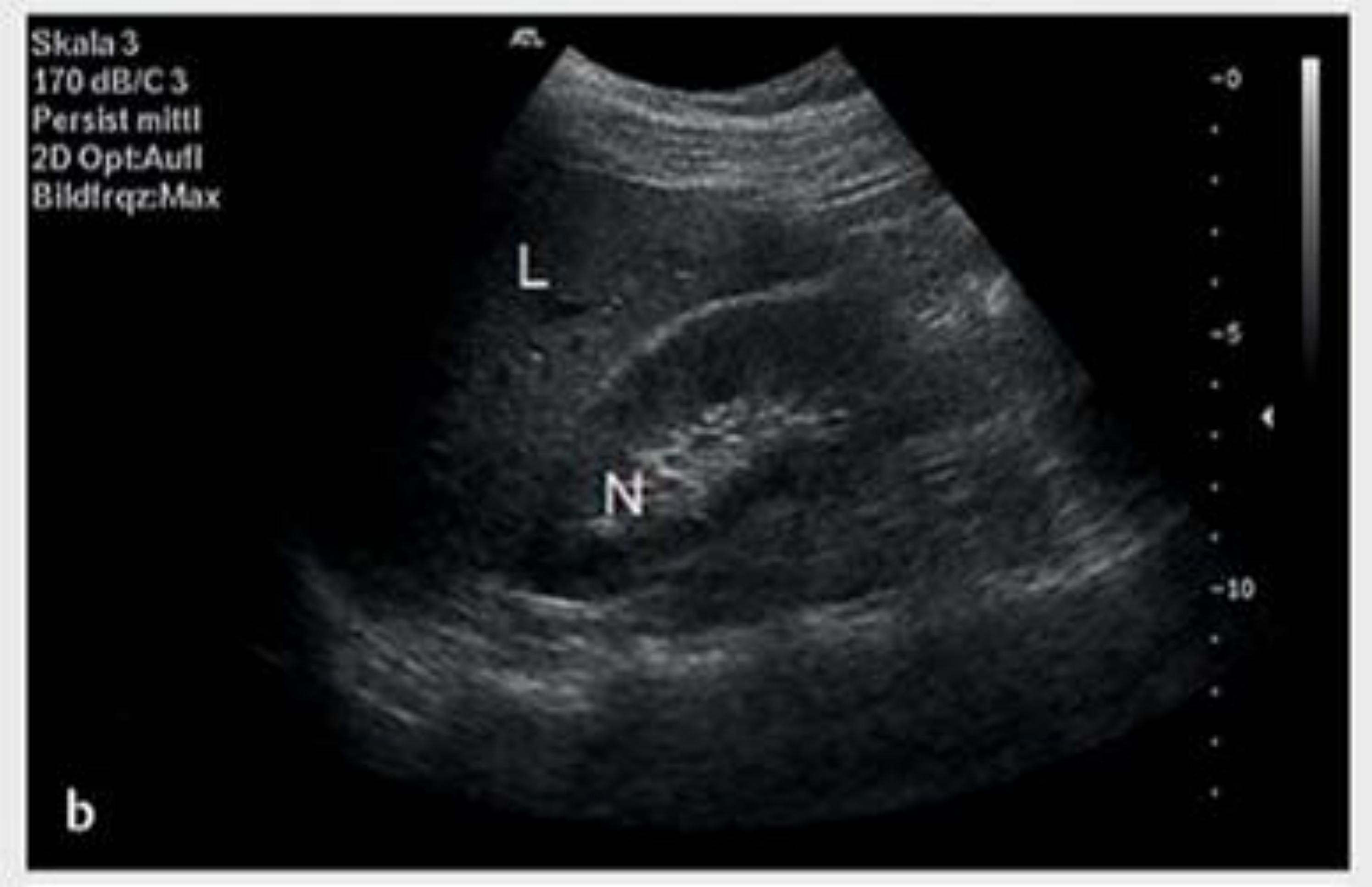

FAST-Position 3: Morison-Pouch. L: Leber, N: Niere. b physiologischer Morison-Pouch ohne Erguss; Quelle: Bord JP et al. Zielorientierte Notfallsonografie bei Traumapatienten (FAST-Protokoll). Dtsch Med Wochenschr 2008; 133: 2646–2648.

MARIEN HOSPITAL HERNE

MARIEN HOSPITAL HERNE
UK RUB UNIVERSITÄTSKLINIKUM DER RUHR-UNIVERSITÄT BOCHUM

Mitten in der
ST. ELISABETH GRUPPE
KATHOLISCHE KLINIKEN RHEIN-RUHR

FAST-Sonographie

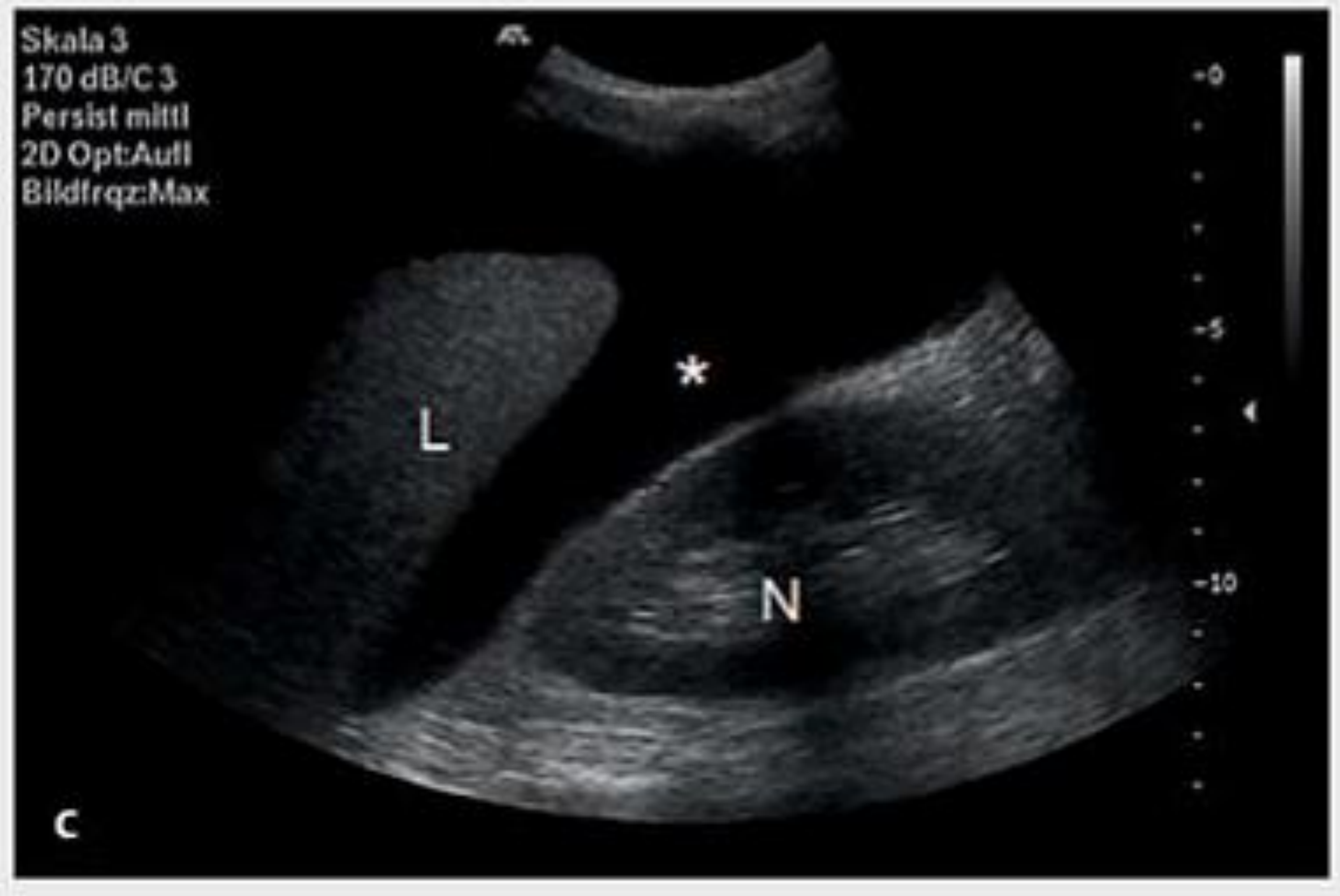

FAST-Position 3: Morison-Pouch. L: Leber, N: Niere. c Pathologischer Morison-Pouch mit Erguss (*); Quelle: Bord JP et al. Zielorientierte Notfallsonografie bei Traumapatienten (FAST-Protokoll). Dtsch Med Wochenschr 2008; 133: 2646–2648.

FAST-Sonographie: Milzverletzungen

- Analog zur FAST-Position 3 zeigt die FAST-Position 5 auf der linken Körperseite den Raum zwischen oberem Nierenpol und Milz, den sog. Koller-Pouch (s. Abb.).
- Hier sammelt sich z. B. nach stumpfem Bauch- oder linksseitigem Thoraxtrauma klassischerweise freie Flüssigkeit.
- Da Niere und Milz etwas kranialer liegen als die Organe der Gegenseite, ist die Diagnostik wegen der durch die Rippen entstehenden Schall-Schatten jedoch manchmal erschwert.

FAST-Sonographie

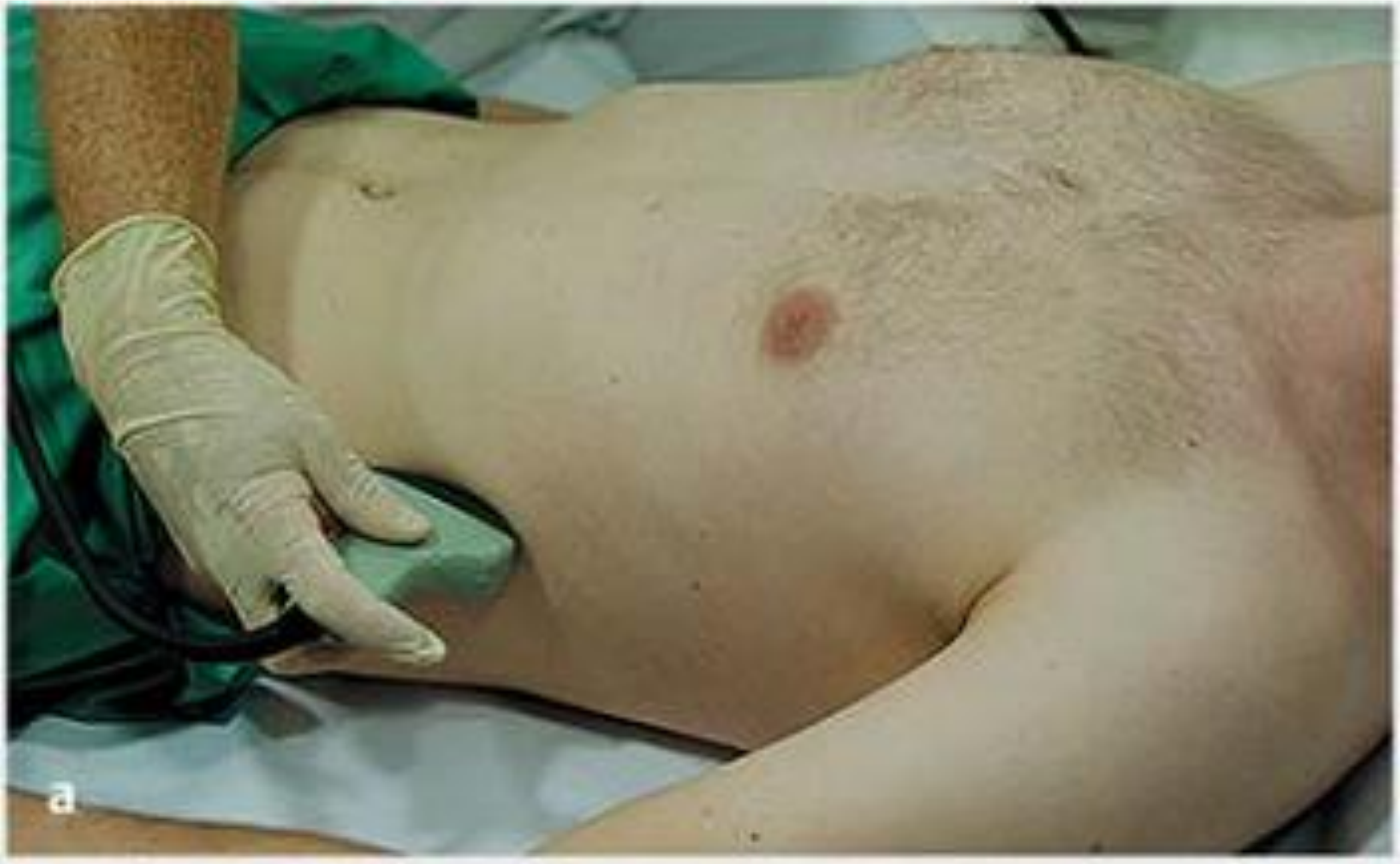

FAST-Position 5: Koller-Pouch. a Darstellung der Schallkopfführung. Quelle: Bord JP et al. Zielorientierte Notfallsonografie bei Traumapatienten (FAST-Protokoll). Dtsch Med Wochenschr 2008; 133: 2646–2648.

MARIEN HOSPITAL HERNE

MARIEN HOSPITAL HERNE
UK RUB UNIVERSITÄTSKLINIKUM DER RUHR-UNIVERSITÄT BOCHUM

Mitten in der
ST. ELISABETH GRUPPE
KATHOLISCHE KLINIKEN RHEIN-RUHR

FAST-Sonographie

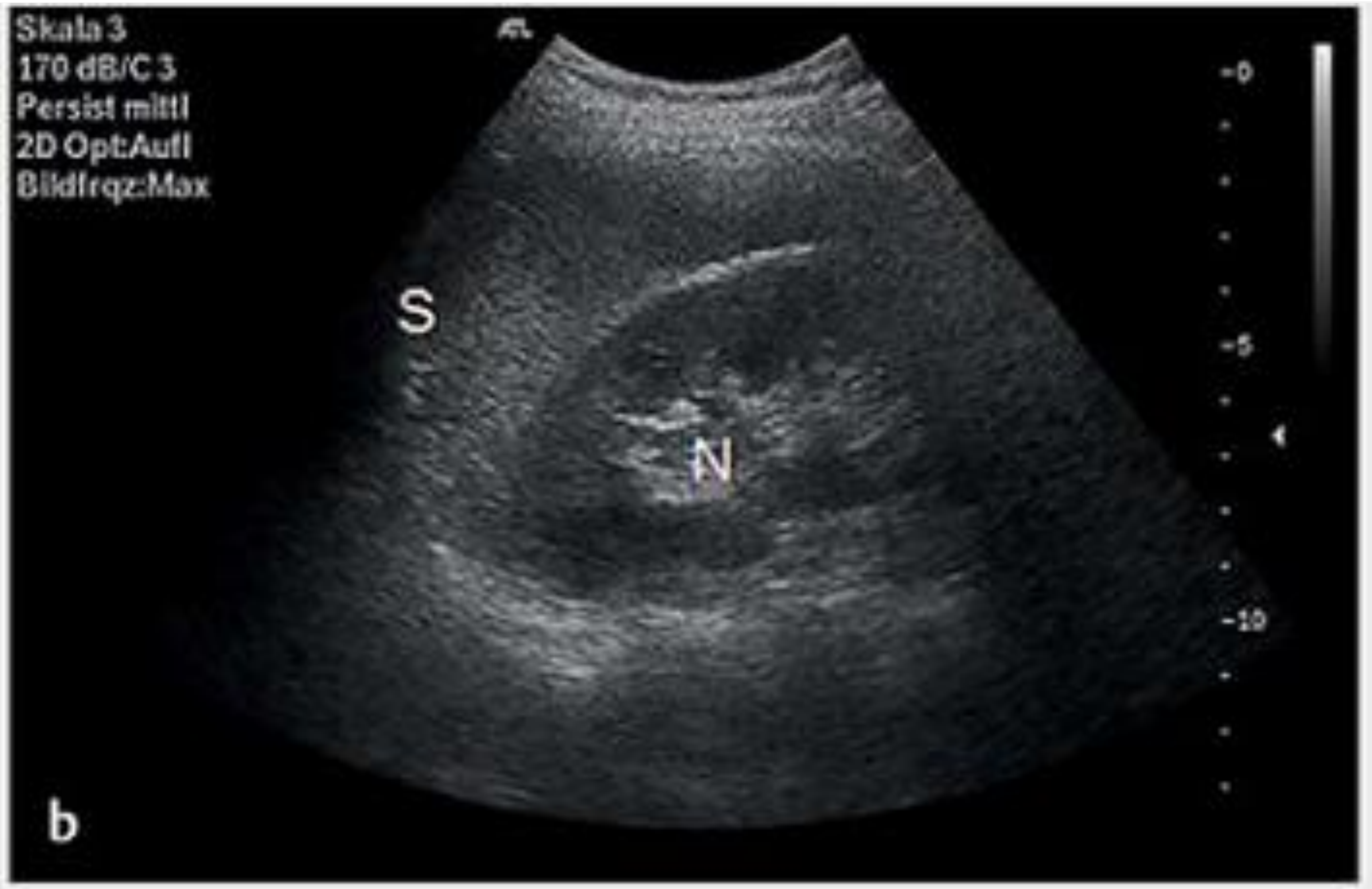

FAST-Position 4: Koller-Pouch. S: Milz, N: Niere. b Physiologischer Koller-Pouch ohne Erguss. Quelle: Bord JP et al. Zielorientierte Notfallsonografie bei Traumapatienten (FAST-Protokoll). Dtsch Med Wochenschr 2008; 133: 2646–2648.

FAST-Sonographie

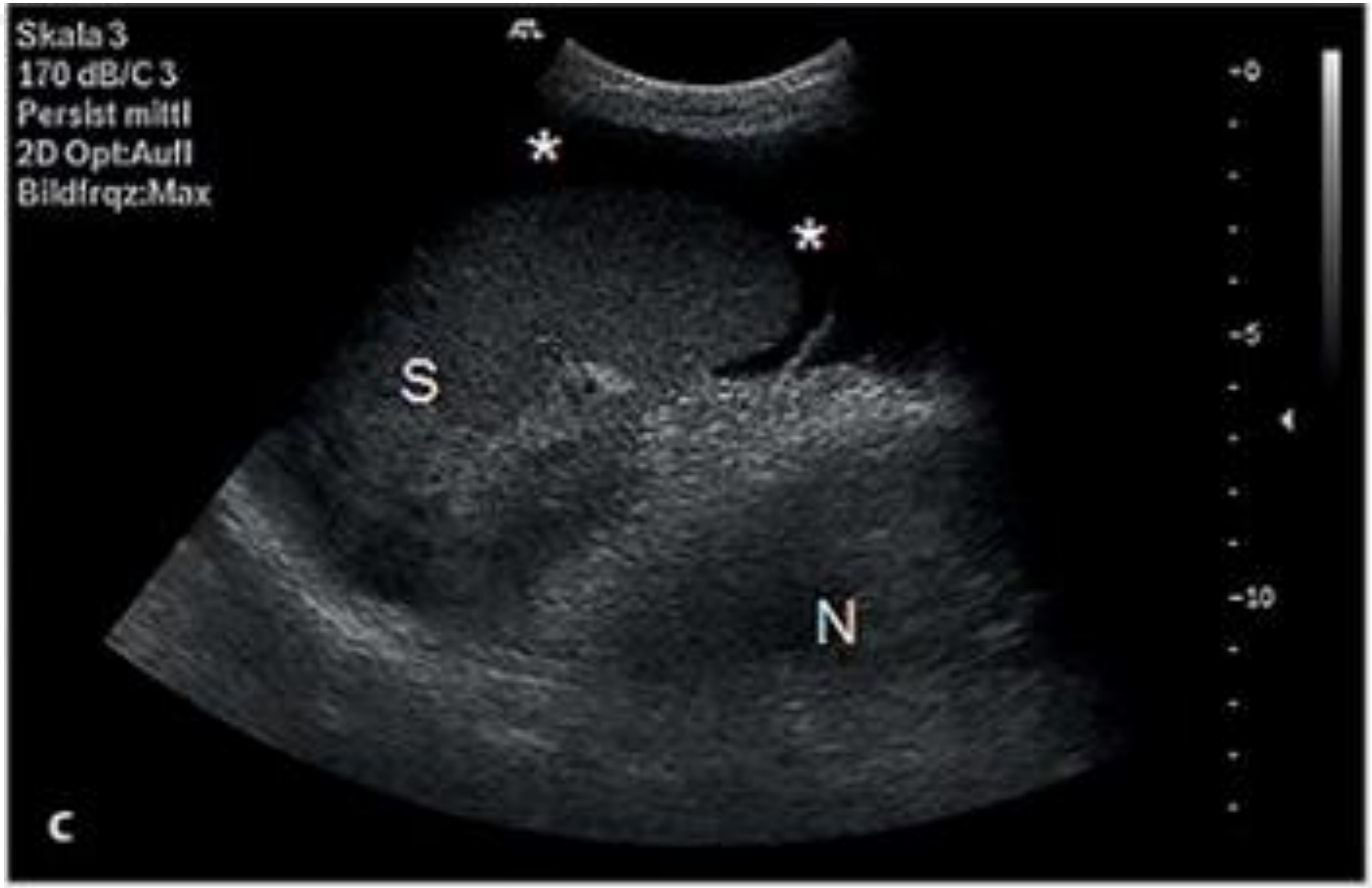

FAST-Position 4: Koller-Pouch. S: Milz, N: Niere. c Pathologischer Koller-Pouch mit Erguss (*). Quelle: Bord JP et al. Zielorientierte Notfallsonografie bei Traumapatienten (FAST-Protokoll). Dtsch Med Wochenschr 2008; 133: 2646–2648.

MARIEN HOSPITAL HERNE
MARIEN HOSPITAL HERNE
UK RUB UNIVERSITÄTSKLINIKUM DER RUHR-UNIVERSITÄT BOCHUM
Mitten in der ST. ELISABETH GRUPPE
KATHOLISCHE KLINIKEN RHEIN-RUHR

FAST-Sonographie: Harnblasenruptur

- Bei Hochrasanztraumen ist auch die Harnblase sehr verletzungsgefährdet. Da Flüssigkeit physikalisch nicht komprimierbar ist, kann eine gefüllte Harnblase in dieser Situation leicht rupturieren.
- Freie intraabdominelle Flüssigkeit sammelt sich der Schwerkraft folgend um die Harnblase.
- Mit der FAST-Position 6 werden bei Frauen der perivesikale und der retrouterine Raum (Douglas-Raum) bei Männern der rektovesikale Spalt (Proust-Raum) sonographiert (s. Abb.). In der Klinik werden beide Räume oft vereinfacht als „Douglas-Raum“ bezeichnet.
- Luftgefüllter Darm → eingeschränkte Sicht
- Gefüllte Blase → gute Schallbedingunen + Orientierung

FAST-Sonographie

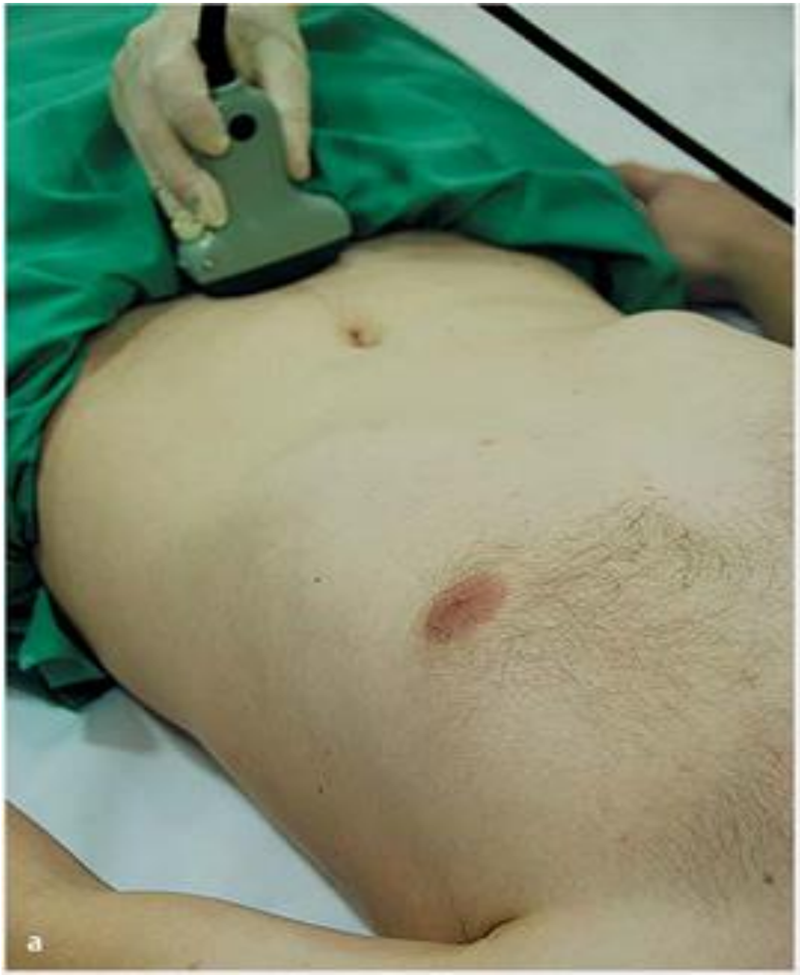

FAST-Position 6: Douglas-Pouch. a Darstellung der Schallkopfführung. Quelle: Bord JP et al. Zielorientierte Notfallsonografie bei Traumapatienten (FASTProtokoll). Dtsch Med Wochenschr 2008; 133: 2646–2648.

FAST-Sonographie

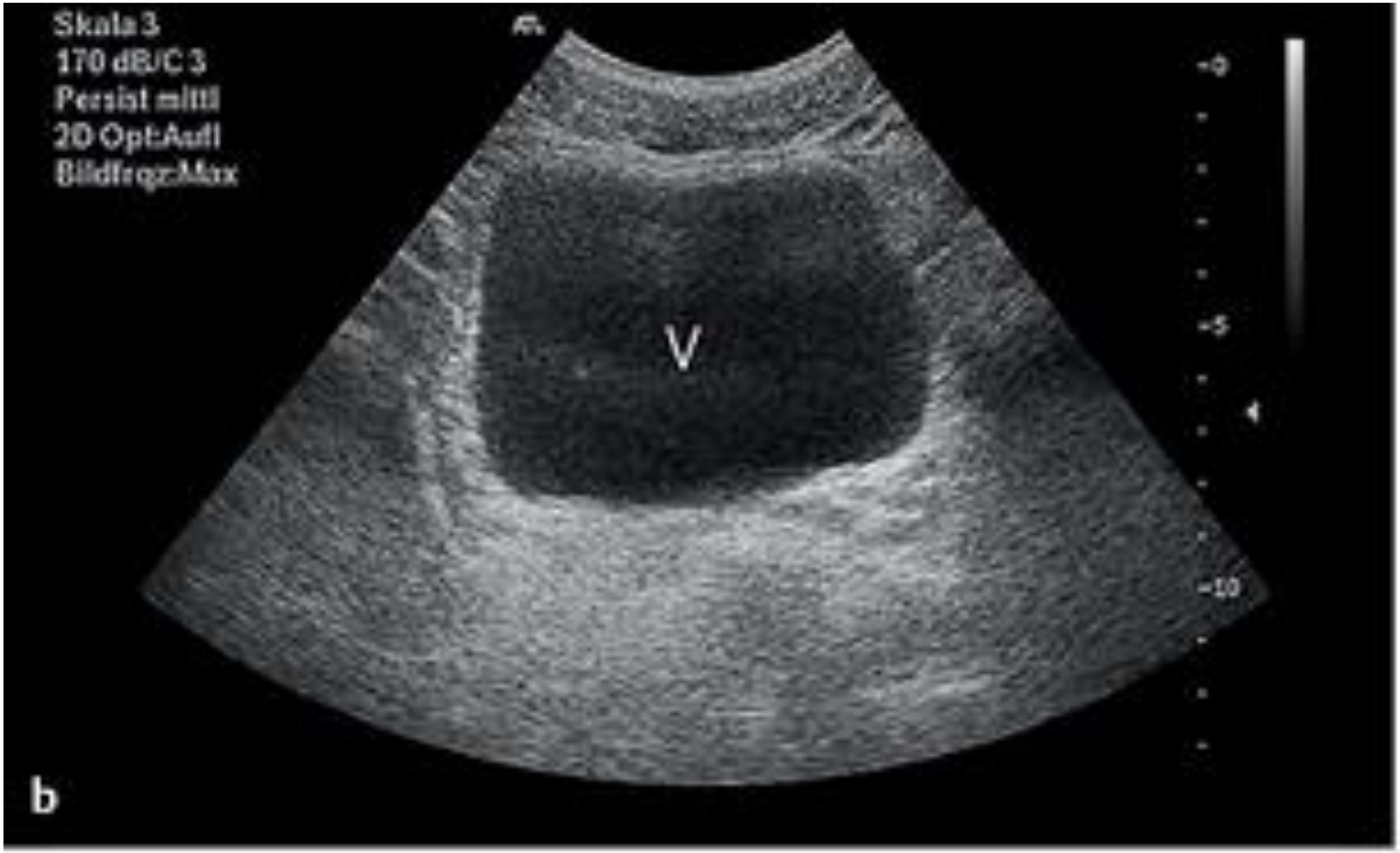

FAST-Position 6: Douglas-Pouch. b physiologischer Douglas-Pouch. Quelle: Bord JP et al. Zielorientierte Notfallsonografie bei Traumapatienten (FASTProtokoll). Dtsch Med Wochenschr 2008; 133: 2646–2648.

MARIEN HOSPITAL HERNE

MARIEN HOSPITAL HERNE
UK RUB UNIVERSITÄTSKLINIKUM DER RUHR-UNIVERSITÄT BOCHUM

Mitten in der
ST. ELISABETH GRUPPE
KATHOLISCHE KLINIKEN RHEIN-RUHR

FAST-Sonographie

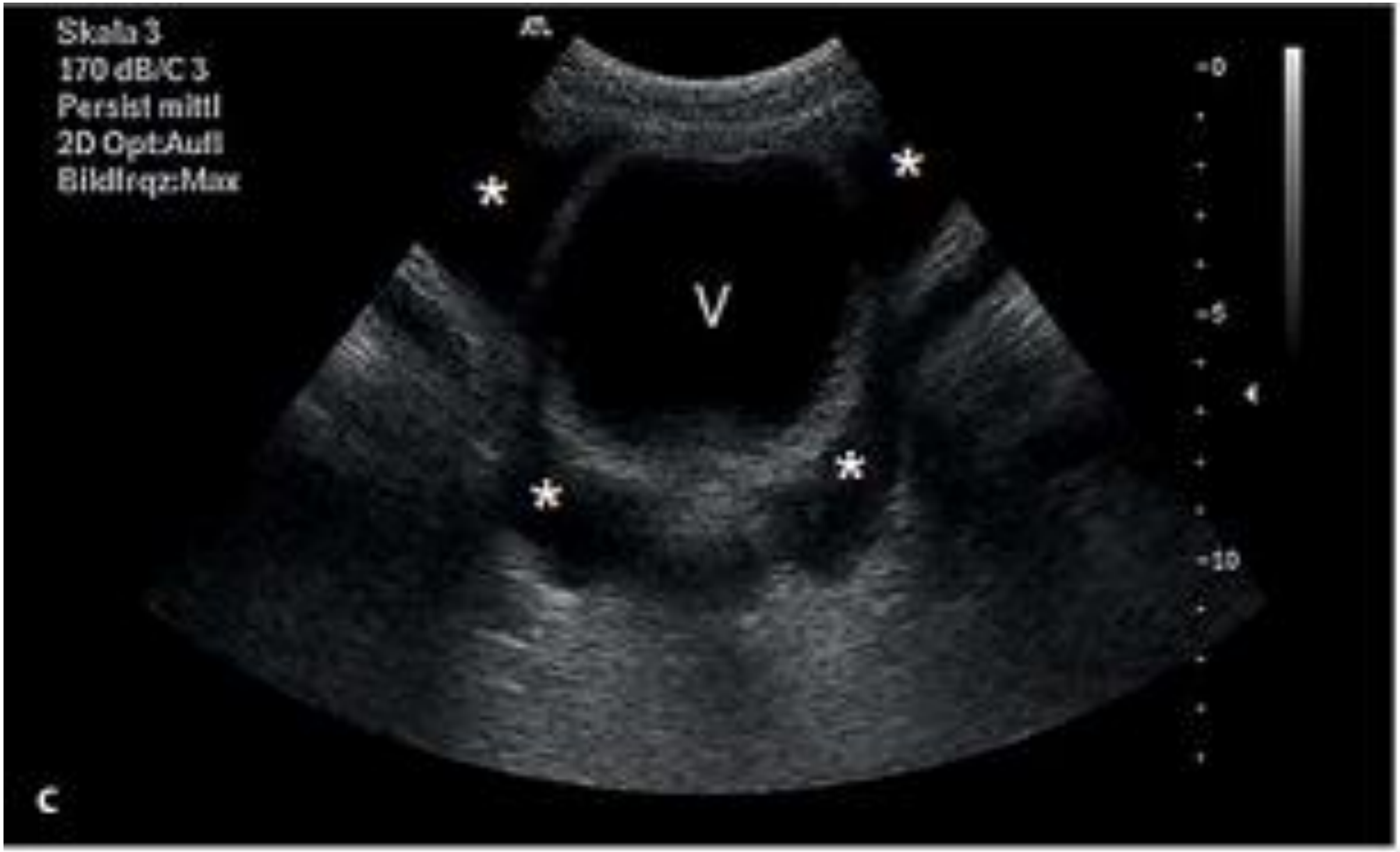

FAST-Position 6: Douglas-Pouch. c Pathologischer Douglas-Pouch mit Erguss (*).

Quelle: Bord JP et al. Zielorientierte Notfallsonografie bei Traumapatienten (FASTProtokoll). Dtsch Med Wochenschr 2008; 133: 2646–2648.

FAST-Sonographie

- <u>Take Home Message:</u>
- Die POC-Sonografie hat in der Notfall- und Intensivmedizin einen extrem hohen Stellenwert.
- Der Konvexschallkopf ist für die abdominelle Sonografie konzipiert.
- Die FAST-Sonografie dient primär dem Nachweis von freier intraabdomineller Flüssigkeit in hämodynamisch instabilen Situationen.
- Darüber hinaus kann sie als Einstieg und Orientierungshilfe für die abdominelle Sonografie gelten

MARIEN HOSPITAL HERNE
MARIEN HOSPITAL HERNE
UK RUB UNIVERSITÄTSKLINIKUM DER RUHR-UNIVERSITÄT BOCHUM
Mitten in der ST. ELISABETH GRUPPE
KATHOLISCHE KLINIKEN RHEIN-RUHR

Durchführung Untersuchungsablauf

- 1.) Untersuchung des Perikards: Anlotung direkt subxyphoidal um den subkostalen Vierkammerblick durchzuführen. Nachweis eines evtl. Perikardergusses, Beurteilung der kardialen Füllung sowie orientierende Einschätzung der Pumpfunktion.
- 2.) Untersuchung des rechten Pleuraspalts und subphrenisch: Die Untersucherhand hat Kontakt zur Liege und wird ca. 5 cm kranial des thorakoabdominellen Übergangs aufgesetzt. Es sollten die kranialen Anteile der Leber sowie das Zwerchfell erkennbar sein. Hier wird der Pleuraspalt auf freie Flüssigkeit (z.B. Blut) untersucht.

Durchführung Untersuchungsablauf

- 3.) Untersuchung des Morison-Pouchs (rechts): Die Hand behält weiterhin Kontakt zur Untersuchungsliege und wird ca. 5cm nach kaudal geschoben, ungefähr auf den thorakoabdominellen Übergang (Höhe der unteren Rippe). Es sollte ein Längsschnitt durch die rechte Niere mit angrenzendem Leberparenchym zu sehen sein.
- 4.) Untersuchung des linken Pleuraspalts und subphrenisch: Die Untersucherhand hat Kontakt zur Liege und wird ca. 5 cm kranial des thorakoabdominellen Übergangs aufgesetzt. Es sollten die kranialen Anteile der Milz sowie das Zwerchfell erkennbar sein. Hier wird der Pleuraspalt auf freie Flüssigkeit (z.B. Blut) untersucht

MARIEN HOSPITAL HERNE

Durchführung Untersuchungsablauf

MARIEN HOSPITAL HERNE

- 5.) Untersuchung des Koller-Pouchs (links): Die Hand behält weiterhin Kontakt zur Untersuchungsliege und wird ca. 5cm nach kaudal geschoben, ungefähr auf den thorakoabdominellen Übergang (Höhe der unteren Rippe). Da die Strukturen auf der linken Seite etwas schwieriger darzustellen sind, sollte der Anlotungspunkt so weit dorsal wie möglich sein. Es sollte ein Längsschnitt durch die linke Niere mit angrenzendem Milzparenchym zu sehen sein.
- 6.) Untersuchung des Douglas-Raums: Beginn im Unterbauchlängsschnitt, Aufsetzen des Schallkopfs direkt kranial der Symphyse in der Medianlinie. Kippen des Schallkopfes nach kaudal, so dass man einen idealen Blick auf Harnblase, Uterus oder Prostata und das Rektum erhält. Der Douglas-Raum wird dann durch Schwenken des Schallkopfs von rechts nach links nach freier Flüssigkeit abgesucht.

FAST-Sonographie: Literatur

- [1] Moore CL, Copel JA. Point-of-care ultrasonography. N Engl J Med 2011; 364: 749–757
- [2] American College of Emergency Physicians. Emergency Ultrasound guidelines. Ann Emerg Med 2009; 53: 550–570
- [3] DGAI Arbeitskreis Ultraschall. AFS-Seminarreihe (Februar 2012). Im Internet: http://www.ak-ultraschall.dgai.de/informationen-und-links/downloads/afs-seminarreihe.html; Stand: 31.10.2018
- [4] DEGUM Arbeitskreis Notfallsonographie. Kurse & Kurscurricula. Im Internet: http://www.degum.de/arbeitskreise/notfallsonographie/kurse-kurscurricula.html; Stand: 31.10.2018
- [5] Dolich MO,McKenney MG, Varela JE et al. 2576 ultrasounds for blunt abdominal trauma. J Trauma 2001; 50: 108–112
- [6] Scalea TM, Rodriguez A, Chiu WC et al. Focused assessment with sonography for Trauma (FAST): Results from an international
- consensus conference. J Trauma 1999; 46: 466–472
- [7] Kirkpatrick AW, Sirois M, Laupland KB et al. Hand-held thoracic sonography for detecting post-traumatic pneumothoraces: The Extend Focused Assessment with Sonography for Trauma(EFAST). J Trauma 2004; 57: 288–295
- [8] Thies K, Gwinnutt C, Driscoll P et al. The European Trauma Course – From concept to course. Resuscitation 2007; 74: 135–141
- [9] American College of Surgeons' Committee on Trauma; ATLS Subcommittee; International ATLS working group. Advanced trauma life support (ATLS®). 9th ed. J Trauma Acute Care Surg 2012; 74: 1363–1366

MARIEN HOSPITAL HERNE

FAST-Sonographie: Literatur

- [10] Körner M, Krötz MM, Degenhart C et al. Current Role of Emergency US in Patients with Major Trauma. Radiographics 2008; 28: 225–242
- [11] Kendall JL, Hoffenberg SR, Smith RS. History of emergency and critical care ultrasound. Crit Care Med 2007; 35: 126–130
- [12] Walcher F, Krisching T, Müller MP et al. Accuracy of prehospital focused abdominal sonography for trauma after a 1-day hands-on training course. Emerg Med J 2010; 27: 345–349
- [13] Greim CA, Roewer N. Transösophageale Echokardiografie für Intensivmediziner und Anästhesisten. 3. Aufl. Stuttgart: Thieme; 2007
- [14] Hahn D, Offermann S, Holmes J. Clinical importance of intraperitoneal fluid in patients with blunt intra-abdominal injury.Am J Emerg Med 2002; 20: 595–600
- [15] Rozycki G, Ochsner M, Feliciano D et al. Early detection of hemoperitoneum by ultrasound examination of the right upper quadrant: a multicenter study. J Trauma 1998; 45: 878–883
- [16] AINS Abdominelle Notfallsonografie in der Intensiv- und Akutmedizin (2019)
