# Supplementary material for: Blended e-learning with handheld ultrasound devices improves practical competence in eFAST: a randomised controlled study
Source: BMC Med Educ. 2026 Mar 25;26:580. doi: 10.1186/s12909-026-09054-5 (PMC13063760; doi:10.1186/s12909-026-09054-5)
Supplement: Supplementary file 3 — Additional file 3. Tools and Systems. [file 12909_2026_9054_MOESM3_ESM.pdf]

Additional file 3: Tools and Systems

- Handheld ultrasound device
  - Model / manufacturer: VScan Air®, GE HealthCare, Chicago, IL, USA.
  - Role in study: Provided to intervention groups for voluntary self-practice during the preparatory phase.
- Ultrasound simulator (assessment)
  - Model / manufacturer: BodyWorks Eve®, Intelligent Ultrasound Ltd., Cardiff, UK.
  - Role in study: Platform for the standardised, video-recorded eFAST examination on the course day.
- Learning management system (digital content delivery)
  - Platform: Moodle (institutional LMS; password-protected access).
  - Role in study: Distribution of the eFAST presentation and instructional video during the 8-day preparatory window.
- Video capture for blinded rating
  - System: qubeAV, SKILLQUBE GmbH, Murg, Germany.
  - Role in study: Recording of simulator examinations for scoring.
- Randomisation tool
  - Service: Randomizer.org (web-based randomisation service), Social Psychology Network, Middletown, CT, USA; https://www.randomizer.org

- Role in study: Generation of the clerkship-group–level allocation sequence with central concealment.
